# Haplotype-resolved genome assembly for tetraploid Chinese cherry (*Prunus pseudocerasus*) offers insights into fruit firmness

**DOI:** 10.1093/hr/uhae142

**Published:** 2024-07-08

**Authors:** Songtao Jiu, Zhengxin Lv, Moyang Liu, Yan Xu, Baozheng Chen, Xiao Dong, Xinyu Zhang, Jun Cao, Muhammad Aamir Manzoor, Mingxu Xia, Fangdong Li, Hongwen Li, Lijuan Chen, Xu Zhang, Shiping Wang, Yang Dong, Caixi Zhang

**Affiliations:** Department of Plant Science, School of Agriculture and Biology, Shanghai Jiao Tong University, Shanghai, 200240, China; Department of Plant Science, School of Agriculture and Biology, Shanghai Jiao Tong University, Shanghai, 200240, China; Department of Plant Science, School of Agriculture and Biology, Shanghai Jiao Tong University, Shanghai, 200240, China; Department of Plant Science, School of Agriculture and Biology, Shanghai Jiao Tong University, Shanghai, 200240, China; Province Key Laboratory, Biological Big Data College, Yunnan Agricultural University, Kunming, Yunnan, 650201, China; Province Key Laboratory, Biological Big Data College, Yunnan Agricultural University, Kunming, Yunnan, 650201, China; Department of Plant Science, School of Agriculture and Biology, Shanghai Jiao Tong University, Shanghai, 200240, China; Department of Plant Science, School of Agriculture and Biology, Shanghai Jiao Tong University, Shanghai, 200240, China; Department of Plant Science, School of Agriculture and Biology, Shanghai Jiao Tong University, Shanghai, 200240, China; School of Materials Science and Engineering, Shanghai Jiao Tong University, Shanghai, 200240, China; Yantai Academy of Agricultural Sciences, Yantai, Shandong, 265500, China; Horticulture Research Institute, Sichuan Academy of Agricultural Sciences, Chengdu, Sichuan, 610066, China; Horticulture Research Institute, Sichuan Academy of Agricultural Sciences, Chengdu, Sichuan, 610066, China; Yantai Academy of Agricultural Sciences, Yantai, Shandong, 265500, China; Department of Plant Science, School of Agriculture and Biology, Shanghai Jiao Tong University, Shanghai, 200240, China; Province Key Laboratory, Biological Big Data College, Yunnan Agricultural University, Kunming, Yunnan, 650201, China; Department of Plant Science, School of Agriculture and Biology, Shanghai Jiao Tong University, Shanghai, 200240, China

## Abstract

Chinese cherry (*Prunus pseudocerasus*) holds considerable importance as one of the primary stone fruit crops in China. However, artificially improving its traits and genetic analysis are challenging due to lack of high-quality genomic resources, which mainly result from difficulties associated with resolving its tetraploid and highly heterozygous genome. Herein, we assembled a chromosome-level, haplotype-resolved genome of the cultivar ‘Zhuji Duanbing’, comprising 993.69 Mb assembled into 32 pseudochromosomes using PacBio HiFi, Oxford Nanopore, and Hi-C. Intra-haplotype comparative analyses revealed extensive intra-genomic sequence and expression consistency. Phylogenetic and comparative genomic analyses demonstrated that *P. pseudocerasus* was a stable autotetraploid species, closely related to wild *P. pusilliflora*, with the two diverging ~18.34 million years ago. Similar to other *Prunus* species, *P. pseudocerasus* underwent a common whole-genome duplication event that occurred ~139.96 million years ago. Because of its low fruit firmness, *P. pseudocerasus* is unsuitable for long-distance transportation, thereby restricting its rapid development throughout China. At the ripe fruit stage, *P. pseudocerasus* cv. ‘Zhuji Duanbing’ was significantly less firm than *P. avium* cv. ‘Heizhenzhu’. The difference in firmness is attributed to the degree of alteration in pectin, cellulose, and hemicellulose contents. In addition, comparative transcriptomic analyses identified *GalAK*-*like* and *Stv1*, two genes involved in pectin biosynthesis, which potentially caused the difference in firmness between ‘Zhuji Duanbing’ and ‘Heizhenzhu’. Transient transformations of *PpsGalAK-like* and *PpsStv1* increase protopectin content and thereby enhance fruit firmness. Our study lays a solid foundation for functional genomic studies and the enhancement of important horticultural traits in Chinese cherries.

## Introduction

**Figure 1:**
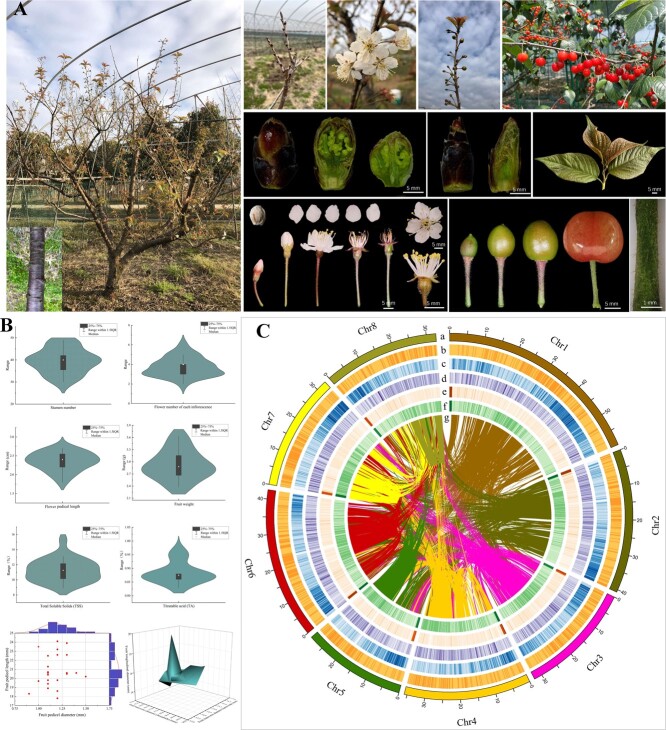
*De novo* genome assembly of *Prunus pseudocerasus*. **A** Phenotypic characteristics of *P. pseudocerasus* trunks, flowers, leaf buds, flower buds, leaves, berries, seeds, and peduncles collected between February and May of 2022 and 2023. **B** Fruit and flower parameters, such as stamen number, flower number of each inflorescence, flower pedicel length, single fruit weight, total soluble solids, titratable acid, fruit pedicel length and diameter, and fruit longitudinal and transverse diameters. **C** Summary of *de novo* genome assembly and sequencing analysis of *P. pseudocerasus.* Moving from outside to inside, the tracks indicate: (a) chromosome size (Mb), (b) GC content, (c) protein-coding gene density, (d) density of DNA transposons, (e) density of LTR/Copia transposons, (f) density of LTR/Gypsy transposons, and (g) synteny blocks among *P. pseudocerasus* chromosomes.

Chinese cherry (*Prunus pseudocerasus*) is one of the most popular stone fruit species in China, with high economic and ornamental value [1]. Its cultivation history in China dates back to 3000–4000 years ago [2]. It originated in southwest China and is now usually cultivated in the temperate zones of the Northern Hemisphere [3, 4]. The plant is characterized by ovate leaves with serrated teeth, corymbose–racemose or umbel inflorescences with two to six flowers, white single suborbicular petals, 30–49 stamens nearly as long as its styles, soft hairs attached to the peduncle and pedicel, and red fruit (Fig. 1A and B; Supplementary Data Table S1). Notably, the fruits are rich in nutrients and trace elements, offering potential preventive benefits against cancer, Alzheimer’s disease, and inflammation-related disorders [5, 6]. The species’ economic importance, potential advantages for human health, and advancing position in Chinese agriculture all contribute to the need for corresponding research that will maintain the global competitiveness of local growers. The haplotype-based chromosome number of *P. pseudocerasus* and other *Cerasus* species is *x* = 8, and the karyotype has been characterized [7–9]. Both wild and cultivated populations of *P. pseudocerasus* are mainly tetraploid [8], indicating that the species has a stable ploidy level.

*Prunus pseudocerasus* cultivars vary widely in phenology, firmness, fruit size and shape, flavor, aroma, texture, antioxidant activity, phenolic composition, and pulp and skin color. Compared with sweet cherry (*P. avium* L.), *P. pseudocerasus* is characterized by earlier blooming and maturation (20–30 days earlier) [10]. Fruit firmness constitutes a critical quality metric for consumers; however, *P. pseudocerasus* exhibits suboptimal performance in this attribute (Supplementary Data Fig. S1). As such, the fruit’s inadequate firmness renders it unsuitable for long-distance transportation, confining its distribution to U-pick operations and local markets—a key constraint on its rapid commercial proliferation throughout China. Conversely, due to its superior fruit firmness, sweet cherry is amenable to global distribution and possesses an extended shelf life, facilitating its worldwide sale. Accordingly, elucidating the reason behind the difference in fruit firmness between *P. pseudocerasus* and *P. avium* holds considerable importance. The processes underlying changes in fruit firmness mainly involve the disassembly of cell wall components, including celluloses, hemicelluloses, and pectins [11, 12]. Pectin is the most complex cell wall component, forming a matrix in which cellulose and hemicellulose are embedded [13]. Degradation occurs via cell wall degrading enzymes, such as pectinases (e.g. polygalacturonases [PGs], pectate lyases [PLs]), xyloglucan endotransglucosylase/hydrolases (XTHs), and β-galactosidase (β-Gal) [14, 15]. Pectinases catalyze different pectins and alter their solubilization, whereas XTHs promote hemicellulose depolymerization [15]. Thus, fruit softening is linked strongly to the expression of genes encoding these enzymes [16, 17]. Pectin biosynthesis requires various nucleotide sugars as activated sugar donor substrates [18]. Galacturonic acid (GalA) is phosphorylated by galacturonic acid-1-phosphate kinase (GalAK) to produce UDP-GalA, which is the precursor for the synthesis of pectins [19]. Glycosyltransferases (GTs) are involved in the biosynthesis of type II arabinogalactan (AG), which is one of the most complex polysaccharides in the cell wall [20]. *Stv1* is a homologous gene of the GT29 family, *AtGALT29A*, which functions in transferring galactose from UDP-galactose to a mixture of numerous oligosaccharides derived from arabinogalactan proteins [21].

The availability of polyploid plant genomes, as exemplified by the tetraploid *Prunus* species, has long been limited by a high degree of heterozygosity, thus impeding research endeavors related to heterosis, desirable traits, and genomic structure. However, technological advancements like third-generation sequencing now allow the assembly of high-quality genomes from an extremely heterozygous genetic background. For instance, chromosome-scale haplotype-resolved genomes of autotetraploid potatoes [22], autopolyploid sugars [23], autotetraploid alfalfa [24], and octoploid strawberries [25] are now available. For *Prunus*, the Chinese plum (*P. mume*) was the first genome to be fully sequenced [26]. Genomes from the subgenus *Cerasus* have also been sequenced, including *P. avium* [27, 28], *P. yedoensis* [29], a *Cerasus* × *yedoensis* hybrid (‘Somei-Yoshino’) [30], *C. serrulata* [31], *P. fruticosa* [32], *P. campanulata* [33], and *P. pusilliflora* [34]. These genomic resources have greatly facilitated our understanding of *Prunus/Cerasus* evolution, origin, and genomic selection. However, *P. pseudocerasus* lacks a chromosome-level genome assembly, hampering efforts to address the fruit’s undesirable lack of firmness. Furthermore, controversy exists over whether they are an autotetraploid or allotetraploid species [8]. Herein, we assembled a chromosome-level haplotype-resolved *P. pseudocerasus* genome using Pacific Biosciences high-fidelity (PacBio HiFi) sequencing, Oxford Nanopore Technology (ONT) sequencing, Illumina sequencing, and high-throughput chromosome conformation capture (Hi-C) technology. We integrated transcriptomics, comparative genomics, and biochemical and functional assays to elucidate the evolution of the *P. pseudocerasus* genome and the diversification of fruit firmness between *P. pseudocerasus* and *P. avium*. The findings of our study offer a valuable resource to facilitate research in the identification of functional genes and molecular breeding in *P. pseudocerasus*.

## Results

### Sequencing, assembly, and annotation of the *P. pseudocerasus* genome

We obtained 98.19 Gb of Illumina short-read data and 83.70 Gb of ONT long-read data (Supplementary Data Table S2). Genome size (1.09 Gb) of *P. pseudocerasus* (PruPse) was estimated using flow cytometry (Supplementary Data Fig. S2; Supplementary Data Table S3). The *P. pseudocerasus* genome was twice the size of the diploid *P. avium* genome (2*n* = 2*x*), suggesting that *P. pseudocerasus* is a tetraploid species, which is consistent with previous reports [35, 36]. After obtaining the draft genome (824.19 Mb) (Supplementary Data Table S4), we conducted a chromosome-level assembly using 176.35 Gb of Hi-C reads. After correcting chromosomal order and orientation, the initial chromosome-level genome assembly (version 1.0, hereafter PruPse V1.0) comprised 154 scaffolds, covering 359.26 Mb, with a contig N50 of 1.11 Mb and a scaffold N50 of 33.00 Mb (Fig. 1C; Supplementary Data Table S5). Within the genome, 305.72 Mb (~85.10%) was anchored to eight pseudochromosomes (Supplementary Data Table S6). The Hi-C heat map did not reveal any notable assembly errors among these eight pseudochromosomes, which were well linked along the diagonal line (Supplementary Data Fig. S3). BUSCO analysis of PruPse V1.0 indicated 97.60% completeness, with only 1.40% missing BUSCOs (Supplementary Data Table S7). We then identified ~158.31 Mb of repetitive sequences, comprising ~51.78% of this genome, which includes simple repeats and transposable elements (Supplementary Data Table S8). The repeat-masked genome served as input data for gene predictors. A total of 41 811 protein-coding genes were identified in PruPse V1.0. The BUSCO completeness between the genome (97.6%) and the annotated gene set (94.1%) was relatively close, implying that most genes in PruPse V1.0 were annotated successfully (Supplementary Data Table S7). Specifically, 41 105 (98.31%) genes were annotated functionally via searches of public protein databases, such as non-redundant (NR), Swiss-Prot, and eggNOG (Supplementary Data Table S9). However, we were unable to assemble the four haplotype genomes of PruPse based on the Illumina and ONT sequencing data.

**Figure 2:**
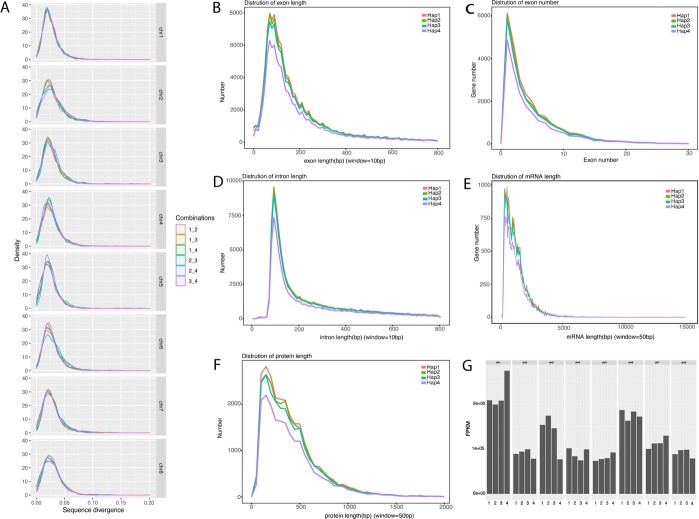
Sequence divergence, gene structure, and expression levels of genes with four alleles from cultivated PruPse. **A** Sequence divergence between allelic chromosomes. Each allelic chromosome was aligned to others using BLAST with default parameters. Sequence divergence was calculated for each alignment block. The distribution of sequence divergence peaks at ~0.02. A comparison of gene structures revealed that exon length (**B**), exon number (**C**), intron length (**D**), mRNA length (**E**), and protein length (**F**) were highly similar across the four haplotype genomes of PruPse. **G** Expression levels of genes with four alleles, presented as fragments per kilobase of exon model per million reads mapped (FPKM) values.

Single-molecule real-time sequencing is more accurate than ONT sequencing. Thus, we conducted PacBio HiFi sequencing using the Sequel II platform, assembling an allele-aware chromosome-level PruPse genome. After filtering low-quality sequences, 42.48 Gb of clean HiFi reads remained, representing 43-fold genome coverage (Supplementary Data Table S10). The PacBio HiFi clean reads were assembled using HiCanu [37], resulting in a 993.69-Mb draft genome with a contig N50 length of 7.05 Mb, and a high value (98.60%) of complete BUSCOs (Supplementary Data Tables S11–S13). Next, we used HiC-Pro and 3D-DNA to scaffold the tetraploid genome (Fig. 2). This algorithm uses Hi-C paired-end reads to build an allele-aware chromosome-scale assembly for polyploid genomes [23]. For each set, we clustered and ordered contigs using Hi-C data to generate eight chromosomes. We investigated the Hi-C contact matrix to determine assembly quality and found that chromosome groups were clearly delineated (Supplementary Data Fig. S4). We then obtained a haplotype-resolved PruPse assembly with four haplotypes of 246.32, 237.05, 225.55, and 192.94 Mb (hereafter Hap1, Hap2, Hap3, and Hap4; Supplementary Data Table S14), containing 97.50, 93.90, 88.50, and 75.10% complete BUSCOs, respectively (Table 1). This effort culminated in the haplotype-resolved genome assembly (PruPse V2.0), encompassing 994.21 Mb across 32 pseudochromosomes and unplaced unitigs, achieving a scaffold N50 of 27.26 Mb, and manifesting 98.60% completeness of BUSCOs. The 32 pseudochromosomes included eight homologous groups each with four allelic chromosomes.

**Table 1:** Metrics of the PruPse genome assembly.

		**PruPse V1.0**	**PruPse V2.0**			
	**Genomic feature**	**ONT**	**Hap1**	**Hap2**	**Hap3**	**Hap4**
	Total assembly size of contigs (Mb)	359 263 463	246 295 535	237 020 056	225 519 133	192 911 129
	Number of contigs	635	65	58	71	69
	Maximum contig length (Mb)	4 247 664	15 118 467	18 125 000	22 750 411	12 967 822
	N50 contig length (Mb)	1 111 332	7 840 664	6 846 412	5 999 568	5 432 551
	N90 contig length (Mb)	304 074	2 153 143	2 247 651	1 578 156	1 451 841
Assembly statistics	L50 contig	104	10	11	10	12
	L90 contig	324	33	33	40	39
	Number of scaffolds	154	8	8	8	8
	N50 scaffold length (Mb)	33 006 389	28 525 531	27 168 469	26 332 636	24 801 833
	Sequence on chromosomes (%)	59 485 093	46 520 301	44 023 726	40 333 243	39 864 160
	Complete (%)	97.60	96.00	93.70	88.60	74.30
	Complete and single-copy (%)	83.50	93.00	90.20	86.00	71.80
Assembly – BUSCO	Complete and duplicated (%)	14.10	3.00	3.50	2.50	2.50
	Fragmented (%)	1.00	0.60	0.70	1.20	1.10
	Missing (%)	1.40	3.40	5.60	10.20	24.60
	Complete (%)	94.1	95.00	93.60	87.20	74.00
	Complete and single-copy (%)	40.9	92.70	90.50	84.70	70.90
Annotation – BUSCO	Complete and duplicated (%)	53.2	2.30	3.10	2.50	3.10
	Fragmented (%)	3.2	1.80	1.50	2.80	2.00
	Missing (%)	2.7	3.20	4.90	10.00	24.00

The long terminal repeat (LTR) assembly index (LAI) serves as a critical metric for assessing the quality of genome assemblies. An analysis of LAI revealed that Hap1 (20.94), Hap2 (20.82), Hap3 (22.0), and Hap4 (21.86) had superior LAI scores compared with previously released *Prunus* genome references [28, 34]. Thus, these four haplotypes are indicative of superior assembly quality (Supplementary Data Table S15). Further analysis, utilizing Repbase (http://www.girinst.org/repbase), revealed that the repetitive sequence ratios in the four haplotypes are comparably high, exceeding 40% (Supplementary Data Table S16). The most abundant transposable elements (TEs) were LTR retrotransposons (LTR-RTs), accounting for 17.56, 17.31, 17.21, and 17.30% of Hap1, Hap2, Hap3, and Hap4, respectively (Supplementary Data Table S16). Most of the LTR-RTs were *Gypsy*/*DIRS1* elements, constituting 10.60, 10.43, 10.52, and 10.05% of Hap1, Hap2, Hap3, and Hap4, respectively, greatly expanding the genome (Supplementary Data Table S16). Among the TEs, DNA transposons accounted for 10.88, 10.86, 11.55, and 10.06% of Hap1, Hap2, Hap3, and Hap4 (Supplementary Data Table S16). Overall, these findings indicate that the expansion of PruPse was caused by TE insertions.

**Figure 3:**
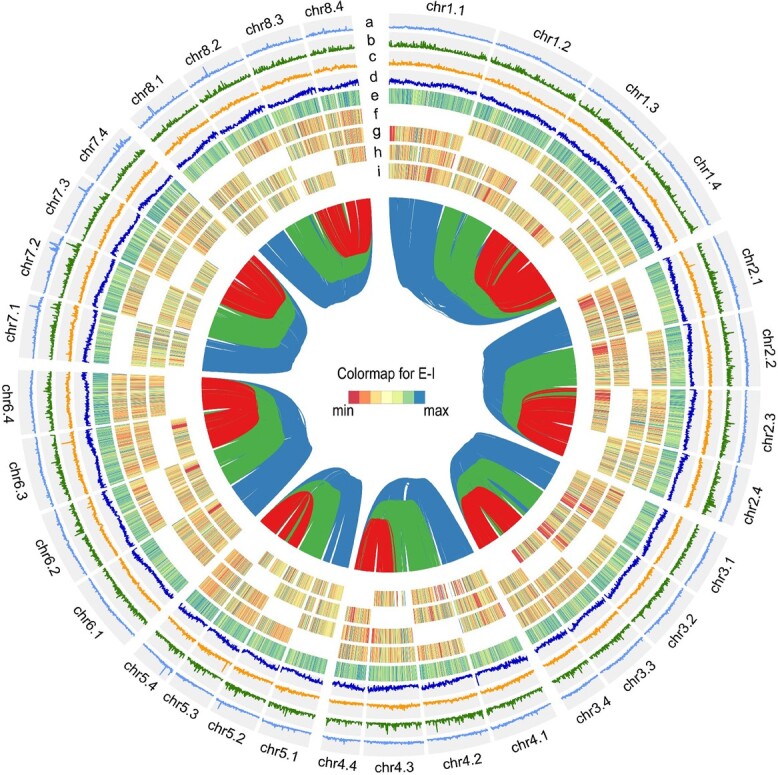
Overview of the haplotype-resolved genome assembly of the ‘Zhuji Duanbing’ (*Prunus pseudocerasus*) genome. From the outermost to the innermost tracks, the following features are indicated: the density of (a) LTR transposons, (b) LINE transposons, (c) DNA transposons, (d) genes; (e) gene expression levels; (f–i) *K*_a_/*K*_s_ ratios of syntenic gene pairs identified between the first (f), second (g), third (h), and fourth (i) haplotype against the other three, respectively. Lines within the core connect synteny blocks, with blue ribbons indicating synteny blocks between the first haplotype and the other three, green ribbons indicating synteny blocks between the second haplotype and the third and fourth, and red ribbons indicating blocks between the third and fourth haplotypes.

Next, we masked repeat regions and annotated protein-coding genes from each monoploid genome. The result was 26 326 protein-coding genes with an average length of 3206 bp, averaging 4.9 exons per gene in the Hap1 genome; 25 289 protein-coding genes with an average length of 3242 bp, averaging five exons per gene in Hap2; 24 213 protein-coding genes with an average length of 3272 bp, averaging five exons per gene in Hap3; and 20 256 protein-coding genes with an average length of 3348 bp, averaging five exons per gene in Hap4 (Supplementary Data Table S17). Complete BUSCOs of gene annotation were 95.0, 93.6, 87.2, and 74.0% for Hap1, Hap2, Hap3, and Hap4, respectively (Supplementary Data Table S18). Using NCBI NR, Swiss-Prot, Gene Ontology (GO), KEGG, and Pfam, the four haplotype genomes contained 25 953 (98.58%), 24 936 (98.60%), 23 897 (98.69%), and 19 967 (98.57%) annotated protein-coding genes, respectively (Supplementary Data Table S19). In addition, 2535 (0.17% of Hap1), 2225 (0.13% of Hap2), 1953 (0.14% of Hap3), and 2186 (0.15% of Hap4) non-coding RNAs (ncRNAs) were predicted, including rRNAs, tRNAs, and miRNAs (Supplementary Data Table S20). We found that the sequence divergence (~0.02) among allelic chromosome pairs was low (Fig. 2A). To clarify whether the *P. pseudocerasus* genome is auto- or allotetraploid, we systematically compared the four allelic chromosomes and found that they were similar in terms of gene structure (Fig. 2B–F). Moreover, gene expression patterns for each allelic chromosome group did not exhibit notable signs of overall allelic dominance (Fig. 2G). Synteny plots and the *K*_a_/*K*_s_ ratios of syntenic gene pairs demonstrated a high degree of conserved synteny, with no notable overall difference in *K*_a_/*K*_s_ ratio observed between any two allelomorphic chromosomes (Fig. 3; Supplementary Data Fig. S5). Thus, *P. pseudocerasus* appears to be a stable random-pairing autotetraploid species, a characteristic that hinders genomic deciphering.

### Synteny and gene family analyses

**Figure 4:**
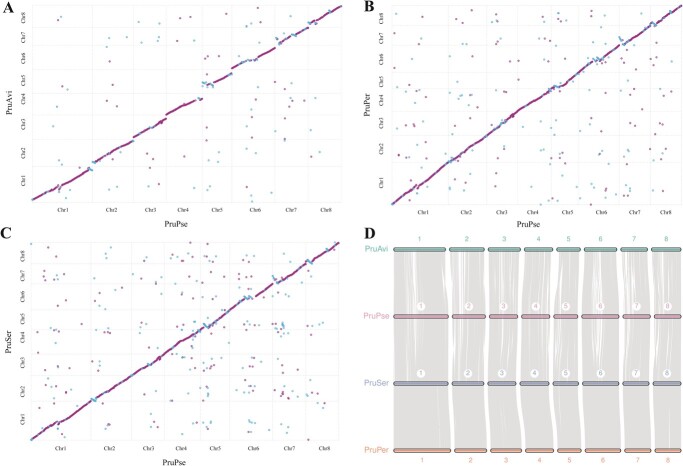
Synteny analysis of *Prunus pseudocerasus*, *P. avium*, *P. serrulata*, and *P. persica*. **A**–**C** Synteny maps between *P. pseudocerasus* and (**A**) *P. avium*, (**B**) *P. persica*, and (**C**) *P. serrulata*. Blue and purple represent similar sequences in the opposite and same orientations, respectively. **D** Syntenic blocks among *P. pseudocerasus*, *P. avium*, *P. serrulata*, and *P. persica*. Each line indicates one block. PruAvi, *P. avium*; PruPse, *P. pseudocerasus*; PruSer, *P. serrulata*; PruPer, *P. persica*. Chr 1–8 = chromosomes 1–8.

**Figure 5:**
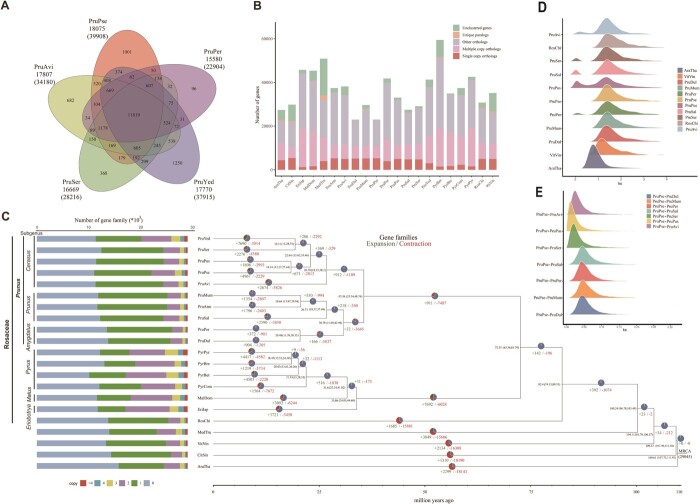
Comparative analysis of gene families between the genomes of *Prunus pseudocerasus* and other species. **A** Venn diagram indicating unique and common gene families among five *Prunus* genomes. **B** Gene number distribution of single- and multiple-copy, and other orthologs, unique paralogs, and unclustered genes in *P. pseudocerasus* (PruPse), *Prunus yedoensis* (PruYed), *Prunus serrulata* (PruSer), *Prunus pusilliflora* (PruPus), *Prunus avium* (PruAvi), *Prunus mume* (PruMum), *Prunus armeniaca* (PruArm), *Prunus salicina* (PruSal), *Prunus persica* (PruPer), *Prunus dulcis* (PruDul), *Pyrus pyrifolia* (PyrPyr), *Pyrus bretschneideri* (PyrBre), *Pyrus betuleafolia* (PyrBet), *Pyrus communis* (PyrCom), *Malus domestica* (MalDom), *Eriobotrya japonica* (EriJap), *Rosa chinensis* (RosChi), *Medicago truncatula* (MedTru), *Vitis vinifera* (VitVin), *Citrus sinensis* (CitSin), and *Arabidopsis thaliana* (AraTha). **C** Phylogenetic tree, divergence times, and outlines of gene families that contracted, expanded, and rapidly evolved in PruPse and 20 other plant species. **D** Synonymous substitution (*K*_s_) for 11 plant species, including PruPse, PruDul, PruMum, PruPer, PruSal, PruSer, PruPus, PruAvi, AraTha, RosChi, and VitVin. **E**  *K*_s_ distribution of orthologous gene pairs from PruPse compared with PruDul, PruMum, PruPer, PruSal, PruSer, PruPus, and PruAvi.

We performed collinearity analyses and constructed three synteny maps after comparing the PruPse genome with the *P. avium* (PruAvi), *P. persica* (PruPer), and *P. serrulata* (PruSer) genomes (Fig. 4A–C). The synteny maps demonstrated that PruPse and the three species have strong collinear relationships with 2757, 6439, and 4147 syntenic blocks, respectively (Supplementary Data Tables S21–S24). A mass of gene syntenic blocks identified by comparing the four *Prunus* genomes were distributed across eight chromosomes, indicating strong cross-species synteny (Fig. 4D). We observed all syntenic blocks that were located on the same chromosome, indicating that *P. pseudocerasus* is closely related to *P. avium* and *P. serrulata* (Supplementary Data Tables S25–S27). Orthologous clustering was performed on the PruPse, PruAvi, PruSer, *P. yedoensis* (PruYed), and PruPer genomes (Supplementary Data Tables S28–S32). We identified 18 075 gene families in PruPse, more than in the other four species (Fig. 5A). The five *Prunus* species shared 11 819 gene families, with PruPse harboring a higher number of unique gene families (1001) than those harbored by PruAvi (682), PruSer (368), and PruPer (96) (Fig. 5A). We subsequently compared the counts of multiple- and single-copy orthologs, unique paralogs, other orthologs, and un-clustered genes between PruPse and 20 selected species (Fig. 5B; Supplementary Data Table S33). Comprehensive statistics on expanded, unique, and contracted gene families of the PruPse genome are presented in Supplementary Data Tables S34–S36. A number of 2229 and 4967 gene families contracted and expanded, respectively, in *P. pseudocerasus* after speciation from *P. avium* (Fig. 5C). The number of expanded gene families was greater in PruPse than that in PruAvi, PruSer, or PruPus. The unique, contracted, and expanded family genes exhibited significant enrichment (*P* < 0.05) across 44, 76, and 218 GO terms, respectively (Supplementary Data Tables S37–S39). Notably, expanded genes showed the most significant enrichment in the ‘cell wall’ term of cellular component (CC) (Supplementary Data Fig. S6), whereas contracted genes exhibited enrichment in the ‘Casparian strip’ term of CC (Supplementary Data Fig. S7).

### Phylogenetic and whole-genome duplication event analyses

To investigate genome evolution, we compared PruPse with 20 other plant species, using *Arabidopsis thaliana* and *Vitis vinifera* as an outgroup. We used 107 high-quality single-copy gene families from 21 plant species to construct a maximum-likelihood phylogenetic tree and found that PruPse was a sister species to PruPus (Fig. 5C). The subgenus *Amygdalus* (PruPer and PruDul) clustered with the subgenus *Prunus* (PruMum, PruArm, and PruSal) and was most closely related to the subgenus *Cerasus* (PruYed, PruSer, PruPus, PruPse, and PruAvi) (Fig. 5C). *Pyrus* was most closely related to *Malus*, followed by *Eriobotrya* (Fig. 5C). The three genera were located on two branches from *Prunus* (Fig. 5C). Based on TimeTree data (http://www.timetree.org/), we assessed when PruPse and other plant species diverged. The split between PruAvi and four *Cerasus* species (PruYed, PruPse, PruPus, and PruSer) was ~26.78 Mya (95% HPD of 18.53–38.20 Mya). The divergence between PruPse and PruPus was estimated to have occurred 18.34 Mya (95% HPD of 12.27–27.44 Mya).

Positively selected gene pairs (*K*_a_/*K*_s_ > 1) for PruPse vs PruAvi, PruPse vs PruSer, and PruPse vs PruPer were identified in quantities of 1724, 1578, and 758, respectively (Supplementary Data Tables S40–S42). We identified 63, 80, and 38 positively selected genes encoding transcription factors (TFs) with matched Pfam domains in PruPse vs PruAvi, PruPse vs PruSer, and PruPse vs PruPer, respectively (Supplementary Data Tables S43–S45). Functional analyses of common TFs, such as NAC, MYB, bHLH, ERF, and bZIP, suggest their involvement in stress response, growth and development, and metabolism in *P. pseudocerasus*. We compared the distribution of synonymous substitution rates (*K*_s_, Fig. 5D) to investigate whole-genome duplication (WGD) events in the PruPse genome. The *K*_s_ distribution of PruPse showed a clear peak at ~1.384, similar to other selected Rosaceae species, indicating that PruPse experienced one common WGD event in the Rosaceae family (Fig. 5D; Supplementary Data Table S46). Referring to the WGD event time in *V. vinifera* (117 Mya) [38], we estimated that the PruPse WGD event arose ~139.96 Mya (Supplementary Data Table S46). We then used *K*_s_ distributions of orthologous genes to deduce divergence of the PruPse with other angiosperms (Fig. 5E). PruPse exhibited a single peak with PruPus, PruSer, PruAvi, PruPer, PruSal, PruDul, and PruMum at a *K*_s_ value of 0.0139, 0.0227, 0.0266, 0.0416, 0.0458, 0.0475, and 0.0485, respectively (Fig. 5E; Supplementary Data Table S47). These values signify the sequential divergence within the subgenera *Cerasus* and *Amygdalus*/*Prunus*, indicating that the diversification among these seven *Prunus* species occurred relatively recently. Additionally, *P. avium* is posited to have diverged earlier than *P. pusilliflora* or *P. serrulata* (Fig. 5E).

### Cell wall degradation is the principal determinant of the soft texture in PruPse compared with PruAvi

**Figure 6:**
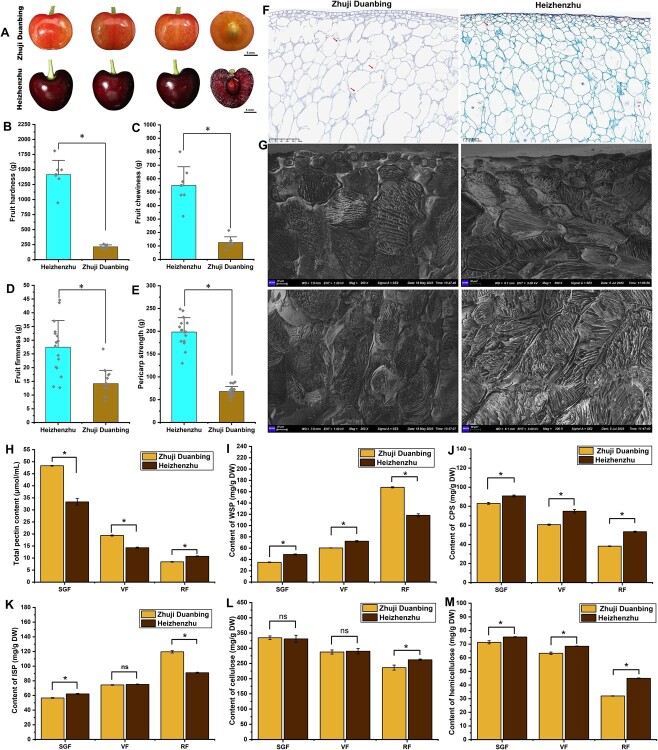
Texture parameters, microstructure, and cell wall components of Chinese cherry (‘Zhuji Duanbing’) and sweet cherry (‘Heizhenzhu’) fruits. **A** Fruit appearance and flesh morphology of ‘Zhuji Duanbing’ and ‘Heizhenzhu’ at the RF stage. Scale bar = 5 mm. **B**–**E** Evaluation of fruit hardness (**B**), chewiness (**C**), firmness (**D**), and pericarp strength (**E**) for ‘Zhuji Duanbing’ and ‘Heizhenzhu’ at the RF stage. **F** Paraffin sections of ‘Zhuji Duanbing’ and ‘Heizhenzhu’ fruits at the RF stage. Red arrows indicate location of cell wall rupture. **G** Cryo-SEM inspection of ‘Zhuji Duanbing’ and ‘Heizhenzhu’ fruits at the RF stage. The upper panels indicate pericarp cells. The bottom panels indicate pulp cells ~1 mm from the pericarp. Sections were isolated from the same location in both varieties. Total pectins (**H**), water-soluble pectins (WSP) (**I**), covalent-soluble pectins (CSP) (**J**), ionic-soluble pectins (ISP) (**K**), celluloses (**L**), and hemicelluloses (**M**) of ‘Zhuji Duanbing’ and ‘Heizhenzhu’ fruits at small green fruit (SGF), veraison fruit (VF), and RF stages. In **B**–**E**, data are presented as the means of at least eight replicates (± standard deviation). In **H**–**M**, data are presented as the means of three biological replicates (± standard deviation). Each replicate comprises five cherry fruits. *P < 0.05, Student’s *t*-test.

We selected our sequenced variety ‘Zhuji Duanbing’ and the sweet cherry cv. ‘Heizhenzhu’ to measure texture-related variables at the ripe fruit (RF) stage (Fig. 6A). ‘Heizhenzhu’ scored significantly higher in texture parameters (hardness and chewiness) than ‘Zhuji Duanbing’ (Fig. 6B and C; Supplementary Data Table S48). ‘Heizhenzhu’ also displayed superior fruit firmness and pericarp strength compared with ‘Zhuji Duanbing’ at this stage (Fig. 6D and E). We also inspected the cytological characteristics of ripe fruit using paraffin sections and cryo-scanning electron microscopy (cryo-SEM). The exocarp cell shape differed between ‘Zhuji Duanbing’ and ‘Heizhenzhu’, the former being square and closely arranged, while the latter was long, oval, and sparse (Fig. 6F and G). ‘Zhuji Duanbing’ displayed greater disruption of pulp cell walls than that displayed by ‘Heizhenzhu’, likely attributable to more pronounced cell wall degradation (Fig. 6F). Subsequently, we analyzed the cell wall components of fruits at the small green fruit (SGF), veraison fruit (VF), and RF stages in both varieties. We observed a decline in total pectin, covalently soluble pectin (CSP), cellulose, and hemicellulose levels, whereas water-soluble pectin (WSP) and ionic-soluble pectin (ISP) levels increased in both varieties during fruit maturation (Fig. 6H–M). Furthermore, the soft-fleshed ‘Zhuji Duanbing’ contained higher pectin levels than the hard-fleshed ‘Heizhenzhu’ at the SGF and VF stages, but lower pectin levels at the RF stage, indicating a sharper decrease in total pectin content for ‘Zhuji Duanbing’ than for ‘Heizhenzhu’ during fruit maturation (Fig. 6H). In the SGF and VF stages, ‘Zhuji Duanbing’ had lower WSP and ISP levels than those exhibited by ‘Heizhenzhu’, but both forms of pectin increased more rapidly in ‘Zhuji Duanbing’ as fruits matured (Supplementary Data Table S49), resulting in WSP and ISP levels at the RF stage being 1.41 and 1.31 times higher, respectively, in ‘Zhuji Duanbing’ than in ‘Heizhenzhu’ (Supplementary Data Table S49). Concurrently, ‘Zhuji Duanbing’ fruits contained less CSP and hemicellulose than ‘Heizhenzhu’ fruits (Fig. 6J and M), with both variables decreasing more sharply during fruit maturation in the former cultivar than in the latter (Supplementary Data Table S49). Cellulose content exhibited a comparable decline in both varieties from the SGF to the VF stage, but dropped to a greater extent in ‘Zhuji Duanbing’ from the VF to the RF stage (Fig. 6L). Overall, these results indicated that fruit softening in the two varieties depends on cell wall degradation, with differences in fruit firmness between the two varieties attributed to varying degrees of alteration in pectin, cellulose, and hemicellulose contents.

### X-ray micro-computed tomography of fruit voids explains firmness variation

**Figure 7:**
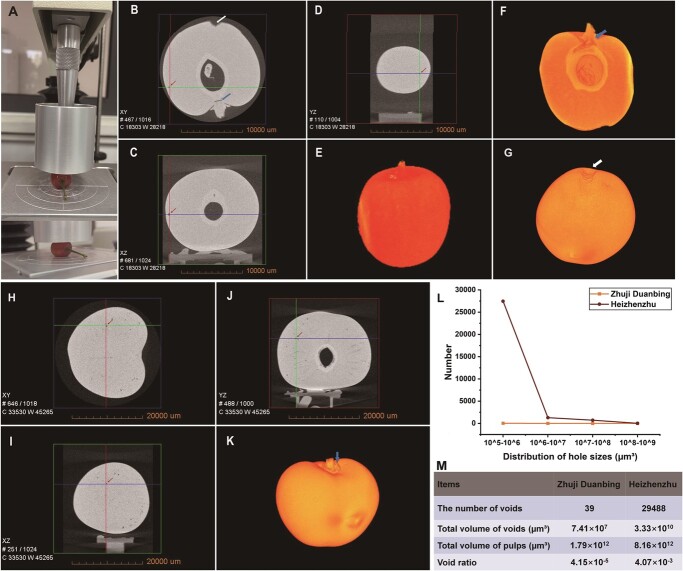
3D visualization of voids in Chinese cherry (‘Zhuji Duanbing’) and sweet cherry (‘Heizhenzhu’) fruits using X-ray micro-CT. **A** Image of fruit extrusion treatment using texture analyzer. **B**–**D** Grayscale images in XY (**B**), XZ (**C**), and YZ (**D**) directions of mature ‘Zhuji Duanbing’ fruits depicting voids (dark areas represent voids and light areas represent organic matter). **E** 3D model of mature ‘Zhuji Duanbing’ fruit. **F** Longitudinal 3D image of rupture at the peduncle base of ‘Zhuji Duanbing’ fruits after extrusion treatment. **G** 3D model of rupture at the top of ‘Zhuji Duanbing’ fruits after extrusion treatment. **H**–**J** Grayscale images in XY (**H**), XZ (**I**), and YZ (**J**) directions of mature ‘Heizhenzhu’ fruits depicting voids. **K** 3D model of mature ‘Heizhenzhu’ fruit. **L** Volume distribution of voids in ‘Zhuji Duanbing’ and ‘Heizhenzhu’ fruits after extrusion. **M** Comparison of total void volume and void proportion between ‘Zhuji Duanbing’ and ‘Heizhenzhu’ fruits. Blue arrow indicates the location of the damaged crack at the peduncle base; white arrow indicates the damaged crack at the top of the fruit; red arrow indicates void location.

X-ray micro-computed tomography (X-ray micro-CT) was developed as a compelling non-destructive tool for 3D imaging in plant anatomy and morphology studies [39–41]. In the present study, we compared the physical structures of ‘Zhuji Duanbing’ and ‘Heizhenzhu’ using X-ray micro-CT technology to clarify the differences in their firmness. Before micro-CT scanning, we first extruded the FR fruits of ‘Zhuji Duanbing’ and ‘Heizhenzhu’ utilizing a texture analyzer (Fig. 7A). The analysis identified numerous low-density voids within ‘Zhuji Duanbing’ and ‘Heizhenzhu’ fruits (Supplementary Data Fig. S8). Large ruptures were present at the peduncle base and tops of ‘Zhuji Duanbing’ fruits (Fig. 7B). Additionally, we found 39 voids in ‘Zhuji Duanbing’ fruits using 2D images (Fig. 7C and D). The rupture characteristics of the wounds were clearly observed after 3D reconstruction (Fig. 7E–G). In addition, using 2D images, we found a dense void mass in ‘Heizhenzhu’ fruits (Fig. 7H–J). Subsequent 3D reconstruction revealed only one rupture at the peduncle base of ‘Heizhenzhu’ fruits and no notable wounds elsewhere (Fig. 7K). We then divided voids into four categories based on their volume: void A (10^8^–10^9^ μm^3^), void B (10^7^–10^8^ μm^3^), void C (10^6^–10^7^ μm^3^), and void D (10^5^–10^6^ μm^3^). ‘Zhuji Duanbing’ had 0 void As, 2 void Bs, 11 void Cs, and 26 void Ds, fewer than the numbers in ‘Heizhenzhu’: 19 void As, 718 void Bs, 1292 void Cs, and 27 459 void Ds (Fig. 7L; Supplementary Data Tables S50 and S51). Unsurprisingly, ‘Zhuji Duanbing’ also had a far lower total void volume (7.41 × 10^7^ μm^3^) than ‘Heizhenzhu’ (3.33 × 10^10^ μm^3^), along with a lower void volume ratio (4.15 × 10^−5^ vs 4.07 × 10^−3^ v/v) (Fig. 7M). These findings underscore the remarkable impact of the pulp texture of cherry on the extent of impact-related pulp damage.

### *GalAK*-*like* and *Stv1* potentially regulate cherry fruit firmness

**Figure 8:**
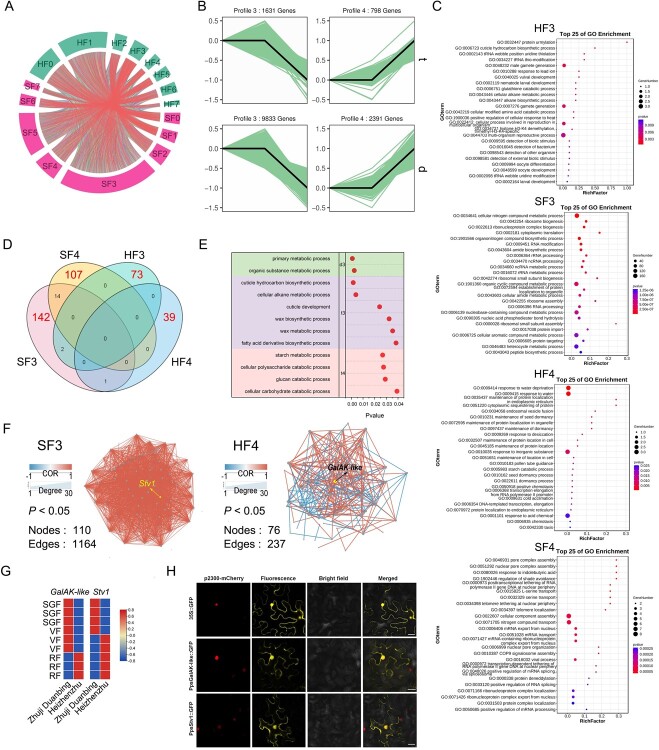
*GalAK-like* and *Stv1* potentially regulate cherry fruit firmness. **A** Expression patterns (EPs) and evolutionary relationship data were combined to identify non-orthologous genes with the same expression pattern. HF, hard-fleshed; SF, soft-fleshed. **B** EP3 and EP4 showed significant differential expression only at the RF stage. **C** GO enrichment of non-orthologs in EP3 and EP4. Color reflects the *P*-values of the GO terms. **D** Venn diagram of specific functions of different EPs in soft-fleshed ‘Zhuji Duanbing’ and hard-fleshed ‘Heizhenzhu’. **E** Specific functions of EP3 and EP4. Color reflects the *P*-values of GO terms. **F** Identification of core members among EP3 and EP4. Color depth indicates the degree of correlation. The size of the symbols denotes network connectivity, where larger symbols signify superior connectivity (*P* < 0.05). Red edges indicate positive correlation, and blue edges indicate negative correlation. **G** Orthologs of *GalAK*-*like* and *Stv1* were significantly expressed in soft-fleshed ‘Zhuji Duanbing’ and hard-fleshed ‘Heizhenzhu’. Colors reflect gene expression level, with redder shades corresponding to higher expression. **H** Subcellular localization of PpsGalAK-like and PpsStv1 proteins in tobacco leaf epidermal cells. 35S::GFP was used as the positive control. Green fluorescence denotes the GFP fusion protein signal; red fluorescence denotes nucleus marker p2300-mCherry. Saffron indicates merged signals. Scale bars = 25 μm.

To better clarify the difference in fruit firmness between ‘Heizhenzhu’ and ‘Zhuji Duanbing, a comparative transcriptomic analysis using SGF, VF, and RF fruits from the two varieties detected 41 811 and 25 720 genes, respectively (Supplementary Data Table S52). We then used the STEM program to calculate differential expression (log_2_ fold change, FC) for all developmental stages relative to the control (SGF). We observed eight expression patterns (EPs) (3^(*n* − 1)^ − 1) for the three developmental stages: EP0–EP7 (Fig. 8A; Supplementary Data Table S53). Subsequent analyses focused on EP3 and EP4 since these genes exhibited differential expression only in RF fruits (Fig. 8B; Supplementary Data Table S53). Next, we used OrthoFinder to obtain orthogroups, orthologs, an orthogroup root gene tree, and all occurrences of gene duplications in the two varieties. Ortholog networks varied in complexity, comprising 2099 different subnetworks with many-to-many, one-to-many, many-to-one, and one-to-one orthogroups (Fig. 8A; Supplementary Data Table S54). GO analysis of non-orthologous EP3 and EP4 genes revealed that they were enriched in several processes, including cell wall modification, cuticle hydrocarbon biosynthesis, and primary metabolic processes (Fig. 8C; Supplementary Data Table S55)*.* Specific functions of different EPs in ‘Zhuji Duanbing’ and ‘Heizhenzhu’ (Fig. 8D) included organic substance metabolic process and primary metabolic process (Fig. 8E; Supplementary Data Table S55). To identify core EP3 and EP4 members, we performed a correlation network analysis. The results showed that *GalAK-like* and *Stv1* had the best connectivity among the genes involved in primary and organic substance metabolic processes (Fig. 8F; Supplementary Data Table S56). The two genes were differentially expressed in the SGF, VF, and RF stages of both ‘Zhuji Duanbing’ and ‘Heizhenzhu’ (Fig. 8G). Subcellular localization using fluorescent protein-tagging then revealed that *PpsGalAK-like* and *PpsStv1* were expressed mainly in the nucleus and cytoplasm (Fig. 8H), consistent with the predictions obtained by CELLO v.2.5.

### *PpsGalAK-like* and *PpsStv1* affect pectin content and thereby change fruit firmness

**Figure 9:**
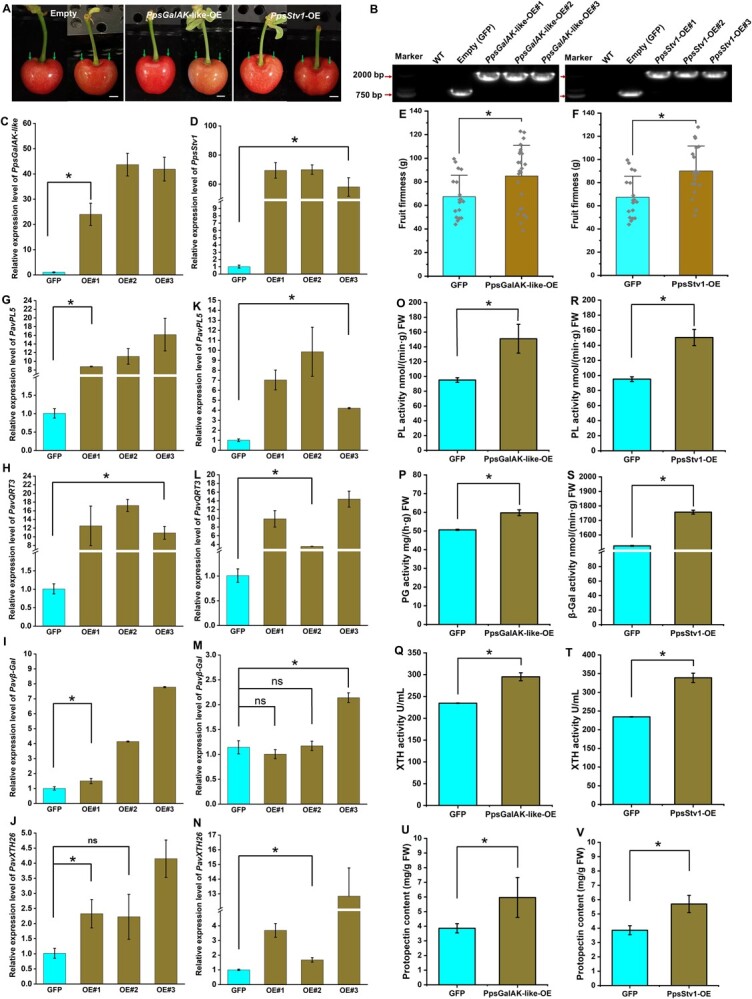
Transient overexpression of *PpsGalAK-like* and *PpsStv1* in cherry fruits. **A** Phenotype of *PpsGalAK-like* and *PpsStv1* transgenic cherry fruits. Green arrows point to injection sites. Scale bar = 5 mm. **B** Confirmation of transformations based on the amplifications of PCR amplicons of the plasmid sequence from extracted genomic DNA. **C**, **D** Relative expression levels of *PpsGalAK-like* (**C**) and *PpsStv1* (**D**) in *PpsGalAK*-*like*-OE and *PpsStv1*-OE transgenic fruits. **E**, **F** Firmness of *PpsGalAK*-*like*-OE (**E**) and *PpsStv1*-OE (**F**) transgenic fruits. **G**–**N** Relative expression levels of *PL5* (G), *QRT3* (**H**), *β-Gal* (**I**), and *XTH26* (**J**) in *PpsGalAK*-*like*-OE transgenic fruits, and of *PL5* (**K**), *QRT3* (**L**), *β-Gal* (**M**), and *XTH26* (**N**) in *PpsStv1*-OE transgenic fruits. **O**–**T** Enzymatic activity of PL (**O**), PG (**P**), and XTH (**Q**) in *PpsGalAK*-*like*-OE transgenic fruits, and of PL (**R**), PG, β-Gal (**S**), and XTH (**T**) in *PpsStv1*-OE transgenic fruits. **U**, **V** Protopectin content in *PpsGalAK*-*like*-OE (**U**) and *PpsStv1*-OE (**V**) transgenic fruits. Data are shown as means ± standard deviation of three biological replicates, each with five fruits. ^*^P < 0.05 (Student’s *t*-test) relative to the empty control (GFP).

We transiently overexpressed *PpsGalAK-like* and *PpsStv1* in cherry fruits to ascertain their function (Fig. 9A). Initial verification of transient transformation in cherry fruits was conducted through PCR amplification and microscopic examination (Fig. 9B). *PpsGalAK-like* expression levels were considerably increased by 24.0-, 43.7-, and 41.9-fold in transgenic fruits *PpsGalAK*-*like*-OE#1, *PpsGalAK*-*like*-OE#2, and *PpsGalAK*-*like*-OE#3, respectively, compared with the control fruits (Fig. 9C). Similarly, *Stv1* expression in the corresponding transgenic fruits was elevated by 69.4-, 69.9-, and 58.0-fold compared with control levels (Fig. 9D). These findings suggest the successful transformation of fruits with the *PpsGalAK-like* and *PpsStv1* overexpression (OE) constructs. Phenotypic characterization revealed that *PpsGalAK*-*like*-OE and *PpsStv1*-OE transgenic fruits had significantly firmer fruits (*P* < 0.05; Fig. 9E and F), implying that *PpsGalAK*-*like* and *PpsStv1* promoted pectin biosynthesis. To determine the genetic mechanism, we measured the expression levels of cell-wall-related genes (i.e. *PL5*, *QRT3*, *β-Gal*, and *XTH26*) in *PpsGalAK*-*like*-OE and *PpsStv1*-OE transgenic fruits. These key pectin metabolism-related genes, *PavPL5*, *PavQRT3*, and *Pavβ-Gal*, were upregulated significantly in *PpsGalAK*-*like*-OE transgenic fruits (*P* < 0.05; Fig. 9G–I). In addition to three pectin metabolism-related genes, *PavXTH26* was more highly expressed in *PpsGalAK*-*like*-OE fruits than in control fruits, with no significant difference observed for *PpsGalAK*-*like*-OE#2 (*P* < 0.05; Fig. 9J). Similarly, *PavPL5*, *PavQRT3*, and *PavXTH26* expression was upregulated in *PpsStv1*-OE transgenic fruits (*P* < 0.05; Fig. 9K, L, and N). Moreover, we observed that *Pavβ-Gal* was upregulated significantly in only *PpsStv1*-OE#3 transgenic fruit (*P < 0.05; Fig. 9M). Investigation of the gene products in *PpsGalAK*-*like*-OE and *PpsStv1*-OE transgenic fruits revealed that PL, PG, β-Gal, and XTH enzymatic activities were upregulated significantly (*P* < 0.05; Fig. 9O–T). Additionally, the protopectin content was increased in *PpsGalAK-like*-OE and *PpsStv1*-OE transgenic fruits, which reflected their fruit firmness (Fig. 9U and V). Overall, these results suggest that *PpsGalAK-like* and *PpsStv1* enhance fruit firmness by improving the protopectin content.

## Discussion

Herein, we report the genome assembly for the Chinese cherry cultivar ‘Zhuji Duanbing,’ the first published genome sequence for *P. pseudocerasus* and the only published *Prunus* tetraploid genome with phased subgenomes. We first generated the PruPse V1.0 genome using Hi-C and ONT technologies, confirming previous research regarding its tetraploid nature [35, 36]. The PruPseV1.0 genome (359.26 Mb) exhibited a larger size than those of the PruYed (323.8 Mb) [29], PruAvi (344.3 Mb) [28], PruSer (265.4 Mb) [31], and *Prunus campanulata* (PruCam) (280.2 Mb) genomes [33], yet was smaller than the *Prunus fruticosa* (PruFru) genome (366.5 Mb) [32]. PruPseV1.0 also showed a repetition rate of 51.78%, surpassing rates observed in PruCam (50.33%) [33], PruSer (49.02%) [31], and PruYed (47.31%) [29], but comparable to the PruFru rate (51.75%) [32], which may explain the large genome size of PruPse V1.0 compared with these species. We also addressed the controversy regarding whether Chinese cherries are autotetraploid or allotetraploid. Our analysis of collinear relationships in the four haplotypes indicated that *P. pseudocerasus* is a stable random-pairing autotetraploid species. Overall, both of the genomes we released are high-quality reference genomes that can be used for further investigation of molecular mechanisms in Chinese cherries.

Phylogenetic analysis revealed that five *Cerasus* species formed a distinct branch with the shortest divergence time, separate from the subgenus *Prunus* species. Consistent with previous findings, PruAvi diverged earlier than PruPus, PruSer, and PruYed [34, 35]. *Prunus pseudocerasus* is more closely related to *P. pusilliflora* than to *P. avium*; this may be due to the fact that *P. pseudocerasus* and *P. pusilliflora* originated from southwest China [34]. Additionally, we estimated that the genus *Prunus* diverged ~35.56 Mya, and the estimated divergence time between PruSer/PruYed and PruAvi was ~26.78 Mya. In contrast, Chin *et al*. [42] and Baek *et al*. [29] estimated that *Prunus* diverged ~56.0 and 66.2 Mya, respectively. Similarly, the age estimates for the split of the subgenera *Prunus* and *Amygdalus* were ~44.0 Mya and the divergence time between PruYed and PruAvi was estimated to be ~35.9 Mya [29]. Recently, Yi *et al*. [31] estimated that *Prunus* diverged ~28.14 Mya, while PruSer/PruYed and PruAvi separated ~21.44 Mya. These discrepancies in estimates may be attributable to the differences in number and species of the plant materials, leading to distinctions in the selected single-copy homologous genes. Alternatively, differences in the timeframes of fossil records used for checksum estimation could also contribute to these discrepancies. Meanwhile, our investigation revealed that *P. pusilliflora*, like other species within the Rosaceae family, underwent a common WGD event. As *P. pusilliflora* is a tetraploid species, we posit that, in addition to the WGD event shared with the Rosaceae family, it has experienced a lineage-specific polyploidy event. However, predicting this event solely based on homologous genes is challenging. We observed minor peaks in regions where the *K*_s_ distribution closely approached zero across several species (PruAvi, PruSer, PruSal, PruPus, and PruPer). These peaks could potentially be attributed to fragmentation or repetition within the genomes of these species (Fig. 5D).

Our research sheds light on the molecular basis of fruit firmness and provides potential targets for improving this desired trait in *P. pseudocerasus*. We observed higher hemicellulose and cellulose contents in hard-fleshed ‘Heizhenzhu’ compared with soft-fleshed ‘Zhuji Duanbing’ at the RF stage (Fig. 6), consistent with the results from other fruits (blueberries and apples) [11, 43]. In addition, our findings revealed that degradation rates of cellulose, hemicellulose, and pectin contributed to firmness differences between Chinese cherry cv. ‘Zhuji Duanbing’ and sweet cherry cv. ‘Heizhenzhu’ (Fig. 6). Notably, however, our results were at odds with a previous report by Zhai *et al*. [44], indicating that only hemicellulose and pectin degradation rates contribute to differences in firmness between soft- and hard-fleshed sweet cherry varieties. This apparent contradiction may be the result of different genetic backgrounds in experimental materials. Regardless, transcriptomic analysis showed that the pectin synthesis genes *GalAK-like* and *Stv1* were differentially expressed between ‘Zhuji Duanbing’ and ‘Heizhenzhu’, strongly suggesting the involvement of pectin content in fruit firmness.

The available technology (Hi-C, HiFi, and allele-aware assembly algorithms) allowed us to assemble all allelomorphic chromosomes of *P. pseudocerasus*, although the features of tetrasomic inheritance may have caused slight errors in phasing all allelomorphic chromosomes. Nevertheless, this haplotype-resolved genome is well assembled and has adequate quality, which can be used in genetic anatomy and molecular breeding of Chinese cherries. In the future, we plan to draw a pan-genome map of *Cerasus* species to further explore the structure variations and copy number variations related to the fruit firmness and flowering periods, which will clarify the role of structure variations in the domestication of *Cerasus* species and help to elucidate their important economic traits. In conclusion, we present a haplotype-resolved genome assembly of Chinese cherry. This is the first step to fully grasping the molecular underpinnings of various desired traits in an economically important *Cerasus* species, although more research on chromosomal structural diversity and the mechanisms of complex allelic expression is required. Nevertheless, our work paves the way for related research in molecular biology, comparative genomics, genetics, and breeding of Chinese cherries.

## Materials and methods

### Plant materials and sequencing

*Prunus pseudocerasus* (Lindl.) G. Don cv. ‘Zhuji Duanbing’ was selected for genome sequencing and assembly. Fruits and leaves were sampled from a 6-year-old ‘Zhuji Duanbing’ tree at Shanghai Jinyuan Fruit and Vegetable Cultivation Professional Cooperative in Shanghai, China (121°24′13″ N, 30°52′18″ E). Stamen number, flower number of each inflorescence, flower pedicel length, fruit weight, total soluble solids, titratable acid (TA), length and diameter of fruit pedicel, and longitudinal and transverse diameters of fruits were determined in this study. The TA value was expressed as percentage of anhydrous malic acid. Genomic DNA was extracted from fresh, young leaves via a DNeasy Plant Mini Kit (Tiangen Biotech Co. Ltd, Beijing, China). The concentration and purity of DNA were assessed using a Nanodrop 2000 spectrophotometer (Thermo Fisher Scientific Inc., Waltham, MA, USA) and Qubit 3.0 (Thermo Fisher Scientific Inc.). After determining DNA integrity via pulsed-field 0.8% agarose gel electrophoresis, samples were separately packaged for Illumina, HiFi, ONT ultralong, and Hi-C sequencing. For Illumina short-read sequencing, several paired-end libraries were constructed using GenElute Plant Genomic DNA Miniprep kits (Sigma–Aldrich Corp., St Louis, MO, USA) as described by the manufacturer on an Illumina HiSeq X Ten platform (Illumina Inc., San Diego, CA, USA) (Supplementary Data Table S57). For long-read sequencing, a Nanopore library was prepared using a PromethION 48 device (Oxford Nanopore Technologies, Oxford, UK) at Novogene Co. Ltd (Beijing, China). For PacBio HiFi sequencing, a standard SMRTbell library was prepared from 50 μg of DNA using the SMRTbell Express Template Prep Kit 2.0 and sequenced on a PacBio Sequel II (Pacific Biosciences, CA, USA). Three Hi-C libraries were constructed via chromatin extraction and digestion, followed by DNA ligation, purification, and fragmentation [45], before sequencing on the Illumina HiSeq X Ten platform. Total RNA was extracted and evaluated as described by Jiu *et al.* [46]. A total of 18 cDNA libraries representing the three fruit samples at the SGF, VF, and RF stages, as well as blooming flower, young leaf and mature leaf of ‘Zhuji Duanbing’, were constructed as described by Quan *et al.* [47]. Transcriptome sequencing was conducted on an Illumina NovaSeq platform. Fruit samples of SGF (10 days after full bloom [DAFB]), VF (25 DAFB), and RF (40 DAFB) stages from ‘Heizhenzhu’ were collected at the cherry orchard of Shanghai Yingzhiyuan Agricultural Technology Co., Ltd (121°32′56″ N, 30°56′45″ E). The detailed analysis procedures followed published descriptions [9]. In addition, six Chinese cherry varieties (‘Zhuji Duanbing’, ‘Xiangxing’, ‘Manaohong’, ‘Zhushahong’, ‘Lixianxiaoyingtao’, and ‘Wenchuanxiaoheiyingtao’) and three sweet cherry varieties (‘Lita’, ‘Daxing’, and ‘Geleisixing’) were collected at the orchard of the Sichuan Academy of Agricultural Sciences (30°46′47″ N, 104°12′30″ E).

### Genome assembly and evaluation

Prior to *de novo* genome assembly, the genome size of PruPse was estimated via flow cytometry (using BD FACScalibur) using tomato as an internal standard. Fastp v.0.20.2 [48] was used to perform the quality control of the next-generation sequencing (NGS) data, including Hi-C reads and whole-genome sequencing paired-end reads, with default parameters to produce clean reads. For the Nanopore data, passed reads were assembled into a *de novo* genome using NECAT v.0.0.1 with default parameters [49]. Subsequently, the assembly underwent three rounds of polishing using Racon with default parameters [50]. All clean NGS genome paired-end reads were performed with two iterations of polishing using Pilon v.1.21 with default parameters [51]. Subsequently, the redundant sequences were removed using purge_dup v.1.2.5 with default parameters, and the final contig genome was produced. The total clean Hi-C data were used to perform chromosome-scale genome assembly using HiC-Pro [52] and 3D-DNA v.180922 [53] with default parameters. Based on the karyotype results [8] and Hi-C heat maps, the chromosome-level genome was manually checked for misorientation using Juicer v.1.6.2 [54]. NGS data were aligned to the assembly using the Burrows–Wheeler Aligner with default settings, yielding an estimate of coverage ratio [55]. For the PacBio Sequel II HiFi reads, the genome was assembled using HiCanu with default parameters [37]. We used the same methods as above for chromosome scaffolding and correction of the resulting genome. Genome integrity was assessed with the LAI, which was calculated using LTR_FINDER v.1.0.7 [56] and LTR_retriever [57]. The completeness and accuracy of the PruPse genome were assessed with BUSCO v.5.3.1 [58].

### Annotation of repetitive sequences

For the ONT-based and HiFi-based genome, we used the same methods. Repetitive elements were predicted using *ab initio* and homology-based predictions. The *ab initio* approach involved extracting complete 3′- and 5′-ends of LTR elements using LTR_FINDER v.1.07 [56], LTRharvest v.1.5.10, and LTR_retriever v.1.8.0 [59] with default parameters. RepeatModeler v.2.0.10 [60] was also used to predict novel repeat elements. The repeat library was downloaded from Repbase v.21.12 [61]. Finally, RepeatMasker v.4.0.7 [62] was used to predict repetitive elements with a *de novo* repeat library and Repbase database. Tandem repeats were annotated using Tandem Repeat Finder (TRF) v.4.09 [63].

### Gene prediction and functional annotation

Protein-coding genes in the PruPse genome were predicted using a combination of *ab initio* and homology- and transcriptome-based methods. We employed Augustus v.3.0.3 [64], SNAP v.2006-07-28 [65], and GlimmHMM v.3.0.1 [66] to conduct *ab initio* gene prediction. We employed the sequences of *Prunus cerasus* (PRJNA295439 and PRJNA327561), *Prunus avium* (PRJNA73727, PRJNA419491, PRJNA595502, and PRJNA550274), and *Prunus subhirtella* (PRJNA596558) to perform homology-based predictions using Exonerate v.2.2.0. Transcriptome-based gene models were then predicted using StringTie v.1.3.4 [67] and PASA [68], utilizing homologous transcriptomes from the Illumina sequencing data (PRJNA260424 and PRJNA1041553). All these data were then integrated using EvidenceModeler v.1.1.1 [69].

Gene functions were predicted by assessing sequence similarity and domain conservation, using the BLAST tool against the NR, KEGG, and Swiss-Prot databases, employing HMMER v.3.0 to search against Pfam and using InterProScan [70] to annotate GO terms. Non-coding RNAs were also predicted in the PruPse genome using tRNAscan-SE v.1.3.1 (tRNA) [71], RNAmmer v.1.2 (rRNA) [72], and INFERNAL v.1.1.2 (miRNA and snRNA) [73]. Other ncRNAs were predicted using Rfam v.1.0.4 [74].

### Sequence divergence, gene structure, and expression level for genes with four alleles

We aligned each allelic chromosome to others using MUMmer v.4.0.0beta2 (parameters: nucmer −g 1000 − c 90 − l 40 − t 6), and calculated the sequence divergence for each alignment block. We also calculated exon length and number, intron, mRNA, and protein length of four haplotypes. The expression levels of genes were quantified in FPKM values using StringTie v.1.3.4, with default parameters. Subsequently, we counted the sum of all gene expression levels on each chromosome of the four haplotypes. Finally, we visualized them using R (https://www.r-project.org/).

### Synteny analysis

To determine genome collinearity, PruPse V1.0 was compared with PruAvi, PruPer, and PruSer using MUMmer v.3.23 (https://sourceforge.net/projects/mummer/files/) with the parameter -i 90 -l 5000. Subsequently, we visualized the analysis results using MUMmer v.3.23. In addition, gene synteny among the eight chromosomes of PruPse, PruAvi, PruPer, and PruSer was determined using DIAMOND v.2.0.7 (https://github.com/bbuchfink/diamond). Syntenic blocks were generated by comparing the PruPse genome against the PruAvi, PruPer, and PruSer genomes using MCScanX with default parameters. The collinearity results were displayed using JCVI (https://github.com/tanghaibao/jcvi).

### Phylogenetic construction, divergence time assessment, and gene family analyses

To identify orthologous genes, the complete genome sequences of *A. thaliana* [75] (AraTha), *Citrus sinensis* [76] (CitSin), *Eriobotrya japonica* [77] (EriJap), *Malus domestica* [78] (MalDom), *Medicago truncatula* [79] (MedTru), *Prunus armeniaca* [80] (PruArm), *Prunus avium* [28] (PruAvi), *Prunus dulcis* [81] (PruDul), *Prunus mume* [82] (PruMum), *Prunus persica* [83] (PruPer), *Prunus pusilliflora* [34] (PruPus), *Prunus salicina* [84] (PruSal), *Prunus serrulata* [31] (PruSer), *Prunus yedoensis* [29] (PruYed), *Pyrus betuleafolia* [85] (PyrBet), *Pyrus bretschneideri* [86] (PyrBre), *Pyrus communis* [87] (PyrCom), *Pyrus pyrifolia* [88] (PyrPyr), *Rosa chinensis* [89] (RosChi), and *Vitis vinifera* [90] (VitVin) were retrieved for comparison with *P. pseudocerasus*. Gene families were identified using OrthoFinder v.2.2.7 (parameters: -t 40 -a 20 -M msa -S diamond -A muscle -T raxml -og). Single-copy ortholog genes were aligned using Muscle, and the alignment results were filtered using Gblocks v.0.91b (parameters: −t = c, −b3 = 8, −b4 = 10, and −b5 = h). A maximum-likelihood phylogenetic tree was constructed using RAxML v.8.2.12 (parameters: -T 20 -f a -N 1000 -m GTRGAMMA -x 123 456 -p 123456 -o VitVin). Divergence time was estimated using the MCMCtree program from the PAML v.4.9j package [91], with known divergence times obtained from TIMETREE software used for calibration. We identified contracted and expanded gene families using CAFÉ v.3.1 [92].

### Positive selection analysis

The ratio of non-synonymous substitutions (*K*_a_ or dN) to synonymous substitutions (*K*_s_ or dS), denoted as ω = *K*_a_/*K*_s_, is considered a reliable method for evaluating evolutionary pressures of protein-coding genes [93]. Single-copy genes from Chinese cherry and three of representative *Cerasus* species were aligned using Muscle, and the alignment results were filtered using Gblocks v.0.91b. We utilized the CodeML program from the PAML v.4.9 h package to deduce the *K*_a_/*K*_s_ ratios [94]. The *K*_a_/*K*_s_ ratio signifies positive selection (ω > 1), neutral evolution (ω = 1), and negative purifying selection (0 < ω < 1) [95]. After identifying positive selection genes, the empirical Bayes method was employed to calculate posterior probabilities and identify positive selection sites [96]. Positive selection genes were subjected to GO and KEGG enrichment analyses using topGO.

### Whole-genome duplication and divergence event analysis

*K*_s_ was applied to investigate WGD and divergence events between PruPse and 10 other plant species. The timeframe of grapevine fossil records was used as a reference to calculate WGD event times of other plant species. We aligned homologous sequences of amino acids using DIAMOND v.2.0.14.152 and subsequently converted them into codon alignments in ParaAT v.2.0. Next, *K*_s_ values were calculated using KaKs_Calculator v2.0 [97]. Collinear blocks from duplication events were classified using median *K*_s_ values between homologous genes.

### Measurement of textural variables

The textural properties of nine randomly selected cherry fruits were assessed using a Texture Analyzer (TA.XT Plus, Stable Micro Systems, UK). The parameter for the texture analyzer was set as follows: P/50 flat probe, pre-test speed of 5 mm/s, post-test speed of 5 mm/s, a pause time between cycles of 5 s, a trigger force of 5 g, and test speed of 0.5 mm/s. The texture parameters of hardness, chewiness, springiness, gumminess, cohesiveness, and resilience were calculated using the built-in software. In addition, 20 fruits were randomly selected for a puncture test to evaluate fruit firmness, with cylindrical probes (P/2) driven 3 mm into the fruit at a compression rate of 0.5 mm/s. Flesh firmness was calculated using the built-in software. The peak force observed during the test was recognized as the pericarp strength. In addition, the fruit firmness of six Chinese cherry and three sweet cherry varieties was determined with a digital firmness tester (TR Turoni, Forlì, Italy). All experiments were conducted at 25 ± 2°C.

### Microstructure assessment of cherry fruits

Paraffin sections were analyzed as described by Xu *et al*. [98]. For cryo-scanning electronic microscopy (cryo-SEM), fruit samples fixed in glutaraldehyde were frozen using liquid nitrogen slush (−210°C) and cryo-fractured in a preparation chamber (−140°C) (Cryo-SEM PP3010 T, Quorum Technologies, UK). Samples were then sublimated (−75°C; 15 min), subjected to platinum metallization (5 mA, 20 s), and observed under a Gemini SEM 300 microscope (Zeiss, Germany).

### Extraction and measurement of cell wall components

Cell-wall materials were extracted from ripe fruits as described previously [99]. Total pectin (Pectins Content Assay Kit, Sangon Biotech, Shanghai, China), WSP (WSP-1-Y, Jiangsu Keming Biotechnology Institute, Suzhou, China), ISP (ISP-1-Y, Jiangsu Keming Biotechnology Institute, Suzhou, China), CSP (CSP-1-Y, Jiangsu Keming Biotechnology Institute, Suzhou, China), cellulose (Cellulose Content Assay Kit, Sangon Biotech), and hemicellulose (Hemicellulose Content Assay Kit, Sangon Biotech) contents were determined in accordance with the manufacturers’ instruction.

### X-ray micro-CT scanning technique used for visualization of fruit voids

Before X-ray micro-CT scanning, RF samples were randomly selected and extruded using a texture analyzer, with cylindrical probes (P/50) driven in at constant 0.5 mm/s and trigger force of 5 g, reaching a depth of 0.3 times the fruit radius. Subsequently, samples were mounted onto a rotary turntable and scanned using a micro-CT scanner (Xradia 520 Versa, Zeiss, USA) equipped with an X-ray source (maximum 120 kV) to get detailed original data on fruit firmness and damage. Only areas larger than three pixels were identified as voids. The number and size of voids in cherry fruits after extrusion were statistically analyzed using Dragonfly.

### Transcriptome analysis

We obtained RNA-seq data derived from SGF (10 DAFB), VF (25 DAFB), and RF (40 DAFB) of ‘Heizhenzhu’ as described in a previous report [100]. Quality control of the RNA-seq reads was performed using FastQC v.0.11.5 with default parameters. We then mapped RNA-seq reads to the genome sequence using HISAT2 v.2.1.0 with default parameters [101]. Differentially expressed genes (DEGs) were determined using DEseq2 v.1.38.3 [102] with a threshold of *P*-value <0.05 and |log_2_ fold change|>1. GO analysis of DEGs was conducted using the GOSeq R package v.3.15. GO terms with a corrected *P*-value <0.05 were considered to be significantly enriched. The functional classification of GO annotation was performed using WEGO to illustrate gene function distribution. For KEGG pathway analysis, DEGs were mapped to KEGG orthologs in the KEGG pathway database (http://www.kegg.jp/kegg/kegg1.html) using blastall [103].

### Examination of non-orthologous genes with the same expression pattern

STEM program v.1.3.13 [104] was utilized to evaluate EPs using transcriptome data from the SGF, VF, and RF stages, with SGF as the control. Clustering of significantly DEGs indicated that 3^(*n* − 1)^ − 1 EPs were theoretically possible. An evolutionary analysis of genes within each EP in soft-fleshed ‘Zhuji Duanbing’ and hard-fleshed ‘Heizhenzhu’ was performed using OrthoFinder v.2.5.4 [105] with the default parameter (e = 10e−3). Quality control procedures were implemented to assess the robustness of the results.

### Correlation network construction and visualization

Correlation networks were established using the R package imsbInfer (https://github.com/wolski/imsbInfer), with relationships determined using Pearson’s rank correlations.

### qRT–PCR

Extracted RNA was reverse-transcribed using a HiScript^®^ III RT SuperMix Kit (Vazyme, Nanjing, China). Then, qRT–PCR was conducted on a LineGene 9600 Plus Fluorescent Quantitative Detection System (FQD-96A, Bioer, Hangzhou, China) using a TB Green™ Premix Ex Taq™ II kit (TaKaRa, Tokyo, Japan). The operating parameters were determined according to the method described by Jiu *et al.* [46]. Relative expression levels of genes were determined using the 2^−ΔΔT^ method as previously described by Livak and Schmittgen [106], with *PavActin1* serving as the reference gene for normalization. The qRT–PCR primer sequences are presented in Supplementary Data Table S58.

### Subcellular localization

The coding sequences of *PavGalAK-like* and *PavStv1* without the stop codon were amplified and inserted into the pHB vector, which harbors two cauliflower mosaic virus (CaMV) 35S promoters, a GFP fluorescent protein tag, and a translation enhancer. This process generated two fusion constructs, namely p35S-*PavGalAK*-*like*-GFP and p35S-*PavStv1*-GFP. Transformation was performed as previously described [107]. Fluorescent protein localization was detected 3–5 days post-infiltration, when GFP fluorescence reached optimal levels, using a confocal laser scanning microscope (Zeiss LSM 780, Germany).

### Transient overexpression in cherry fruits

Transient overexpression was conducted following previously established methods [44], with slight modifications. The full-length coding sequences of *PavGalAK*-*like* and *PavStv1* were amplified and inserted into the pHB vector and transformed into *Agrobacterium tumefaciens* GV3101. Approximately 200 μl of *A. tumefaciens* suspension containing *PavGalAK*-*like*/*PavStv1* and the control empty vector were then separately injected into the cherry fruits by injecting it until the entire fruit was infiltrated. Approximately 20 fruits were permeated with each gene. The inoculated cherry fruits were harvested 1 week after infiltration, and 15 fruits were collected after discarding malformed ones for subsequent analyses. Genomic DNA was extracted using the CTAB method [108]. The primer sequences for transient overexpression assay are presented in Supplementary Data Table S58.

### Determination of enzymatic activity

After extracting samples with a plant physiology kit, the enzyme activities of pectate lyase (PL, Shanghai Fusheng Industrial Co., Ltd, Shanghai, China), polygalacturonase (PG, Shanghai Fusheng Industrial Co., Ltd), β-galactosidase (β-Gal, Shanghai Fusheng Industrial Co., Ltd), and xyloglucan endotransglucosylase/hydrolase (XTH, Shanghai Fusheng Industrial Co., Ltd) were determined using a microplate reader (Infinite^®^ M1000 Pro, Tecan Group Co., Ltd, Switzerland) in accordance with the manufacturer’s instructions.

### Statistical analysis

All data were analyzed using SAS software v.9.3 (SAS Institute Inc., Cary, NC, USA). Statistical differences were determined using a two-tailed Student’s *t*-test. The results of fruit texture parameters are presented as the means of at least eight replicates (± standard deviation). For qRT–PCR, cell wall components, and enzyme activity assays, data are presented as the means of three replicates (± standard deviation), with each replicate consisting of five fruits. Significance was set at *P* < 0.05.

## Acknowledgements

This work was funded by the China Agriculture Research System (Grant No. CARS-30-2-08), and the Natural Science Foundation of Shanghai (23ZR1430600). We thank Dr Zongyi Sun from Wuhan Grandomics Bioscience Co., Ltd for providing valuable advice.

## Author contributions

C.Z. and Y.D. conceived and designed the experiments; S.J. performed the main experiments and sequencing data analysis, and drafted the manuscript; S.J. and B.C. performed the assembly and annotations; Z.L., H.L., and Y.X. measured textural variables; S.J., F.L., X.Z., and L.C. collected the samples; S.J. and Y.X. worked on the phenotyping; M.L. performed comparative transcriptomic analysis; S.J. performed X-ray micro-CT scanning; J.C. and M.X. performed visualization analysis of fruit voids; S.J. and Z.L. performed the statistical analysis; M.A.M., X.D., and S.W. participated in discussions and provided some valuable advice. All authors provided final approval for publication.

## Data availability

The raw genome sequencing data of *Prunus pseudocerasus* are available at the National Genomics Data Center (https://ngdc.cncb.ac.cn/) under BioProject number PRJCA010538 (CRA013614). The raw read data of transcriptome sequencing were uploaded to the NCBI Sequence Read Archive (SRA) database with accession number PRJNA1041553. All data are available from the corresponding author upon request.

## Conflict of interest

The authors declare no conflicts of interests.

## Supplementary data

Supplementary data are available at *Horticulture Research* online.

## Supplementary Material

Web_Material_uhae142
