# Supplementary material for: Haplotype-resolved genome assembly for tetraploid Chinese cherry (Prunus pseudocerasus) offers insights into fruit firmness
Source: Hortic Res. 2024 Jul 8;11(7):uhae142. doi: 10.1093/hr/uhae142 (PMC11233885; doi:10.1093/hr/uhae142)
Supplement: Web_Material_uhae142 [file web_material_uhae142.zip › Supplementary Tables-revised/Table S1.docx]

**Table S1. Phenotypic characteristics of flower and fruit organs in *Prunus pseudocerasus*.**

| No. | Stamen number | Flower pedicel length  (cm) | Flower number of each inflorescence | Fruit pedicel length/mm | Fruit pedicel diameter /mm | Titratable acid (TA)（%） | Total soluble solids (%) | Fruit longitudinal diameter (mm) | Fruit transverse diameter (broadside) (mm) | Fruit transverse diameter (narrowside) (mm) | Fruit weight (g) |
| --- | --- | --- | --- | --- | --- | --- | --- | --- | --- | --- | --- |
| 1 | 39 | 2.7 | 5 | 19.8 | 1.2 | 0.87 | 9.5 | 15.5 | 17.3 | 15.5 | 3.12 |
| 2 | 41 | 1.8 | 4 | 18.6 | 1.2 | 0.87 | 11.5 | 15.3 | 19.2 | 14.5 | 2.37 |
| 3 | 47 | 2.3 | 4 | 19.7 | 1.0 | 0.96 | 10.7 | 15.5 | 18.4 | 14.4 | 2.37 |
| 4 | 39 | 2.6 | 3 | 23.2 | 1.2 | 0.88 | 10.1 | 16.0 | 16.5 | 14.3 | 2.88 |
| 5 | 30 | 2.7 | 2 | 23.9 | 1.3 | 0.94 | 11.8 | 14.5 | 16.9 | 14.4 | 2.64 |
| 6 | 33 | 2.4 | 3 | 21.3 | 1.1 | 0.88 | 12 | 15.6 | 17.1 | 15.3 | 3.63 |
| 7 | 32 | 2.5 | 3 | 20.6 | 1.4 | 0.86 | 12.3 | 15.1 | 17.2 | 14.3 | 3.64 |
| 8 | 48 | 2.1 | 5 | 20.2 | 1.5 | 0.88 | 10.2 | 15.8 | 16.4 | 15.3 | 2.77 |
| 9 | 42 | 2.2 | 4 | 20.7 | 1.1 | 0.86 | 10.5 | 19.7 | 16.5 | 14.9 | 2.64 |
| 10 | 35 | 2.0 | 6 | 20.6 | 1.3 | 0.83 | 12.5 | 15.6 | 16.9 | 14.6 | 2.72 |
| 11 | 41 | 2.3 | 3 | 23.5 | 1.0 | - | 8.9 | 16.5 | 17.6 | 15.6 | 2.85 |
| 12 | 36 | 2.6 | 4 | 22.6 | 1.2 | - | 9.8 | 15.1 | 17.0 | 14.4 | 2.92 |
| 13 | 41 | 2.2 | 3 | 22.5 | 1.1 | - | 12.0 | 16.7 | 18.8 | 15.6 | 2.93 |
| 14 | 49 | 2.0 | 4 | 24.1 | 1.2 | - | 9.6 | 15.8 | 17.0 | 14.4 | 3.12 |
| 15 | 41 | 2.4 | 3 | 20.5 | 1.3 | - | 11.6 | 15.9 | 17 | 15.6 | 2.69 |
| 16 | 38 | 2.5 | 3 | 20.6 | 1.1 | - | 10.5 | 16.2 | 17.6 | 14.9 | 2.38 |
| 17 | 42 | 2.4 | 5 | 19.8 | 1.1 | - | 10.2 | 16.8 | 19.0 | 15.5 | 2.88 |
| 18 | 32 | 2.4 | 3 | 18.9 | 1.1 | - | 13.1 | 16.1 | 17.5 | 15.0 | 2.64 |
| 19 | 47 | 2.3 | 5 | 17.8 | 1.2 | - | 11.3 | 16.6 | 18.6 | 15.6 | 3.42 |
| 20 | 38 | 2.6 | 3 | 22.6 | 1.3 | - | 11.2 | 16.1 | 16.5 | 14.5 | 2.76 |
| 21 | - | - | 4 | 19.4 | 1.1 | - | 13.0 | 15.6 | 17.5 | 14.7 | 3.19 |
| 22 | - | - | 4 | 18.3 | 0.9 | - | 15.3 | 15.3 | 16.6 | 13.7 | 3.23 |
| 23 | - | - | 3 | 20.4 | 1.1 | - | 12.9 |  | - | - | 3.39 |
| 24 |  | - | - | 19.7 | 1 | - | 9.4 | - | - | - | 2.95 |
| 25 | -- | - | - | - | 1.2 | - | - | - | - | - | - |
| AVG | - | 2.35 | - | 20.80 | 1.17 | 0.88 | 11.25 | 15.97 | 17.41 | 14.86 | 2.92 |
