# Supplementary material for: Haplotype-resolved genome assembly for tetraploid Chinese cherry (Prunus pseudocerasus) offers insights into fruit firmness
Source: Hortic Res. 2024 Jul 8;11(7):uhae142. doi: 10.1093/hr/uhae142 (PMC11233885; doi:10.1093/hr/uhae142)
Supplement: Web_Material_uhae142 [file web_material_uhae142.zip › Supplementary Tables-revised/Table S2-S15-revised.docx]

**Table S2. Data statistics of whole-genome sequencing for *Prunus pseudocerasus.***

| Libraries | Total data (bp) | Read length (bp) | N50 (bp) | Coverage fold |
| --- | --- | --- | --- | --- |
| Illumina | 98,187,578,100 | 150 | - | ~89.26 |
| Nanopore | 83,701,413,578 | - | 30,031 | ~76.09 |
| Hi-C | 176,352,637,500 | 150 | - | ~160.32 |
| Total | 400,720,173,445 | - | - | - |

**Table S3. The estimation of genome size of Chinese cherry cv. ‘Zhuji Duanbing’ using flow cytometry.**

| Sample code | Internal reference species | Fluorescence intensity of tomato | Fluorescence intensity of C6 | Ratio | Genome size (Gb) |
| --- | --- | --- | --- | --- | --- |
| C6 | Solanum lycopersicum | 26.07 | 15.85 | 0.61 | 1.09 |
| C6 | Solanum lycopersicum | 25.84 | 15.81 | 0.61 | 1.10 |
| C6 | Solanum lycopersicum | 25.69 | 15.62 | 0.61 | 1.09 |

**Table S4. Statistical results of contig genome assembly for *Prunus pseudocerasus.***

| Sample ID | Contig | |
| --- | --- | --- |
|  | Length (bp) | Number |
| Total | 824,190,195 | 2,293 |
| Max | 4,247,664 | - |
| N50 | 868,930 | 290 |

Note: N50 refers to the size above which 50% of the total length of the sequence assembly can be found.

**Table S5. Statistical results of the chromosome-level genome assembly of PruPse V1.0**

| Sample ID | Scaffold | | Contig | |
| --- | --- | --- | --- | --- |
|  | Length(bp) | Number | Length(bp) | Number |
| Max | 59,485,093 | - | 4,247,664 | - |
| N50 | 33,006,389 | 5 | 1,111,332 | 104 |
| Total | 359,263,463 | 154 | 359,022,962 | 635 |

Note: N50 refers to the size above which 50% of the total length of the sequence assembly can be found.

**Table S6. Data statistics for ordering and orienting the scaffolds on 8 pseudomolecules.**

| Chromosome | Length (bp) | N_base | Gap_ratio (%) |
| --- | --- | --- | --- |
| PAV_r1.0chr1 | 59,485,093 | 48500 | 0.08 |
| PAV_r1.0chr2 | 40,680,104 | 25500 | 0.06 |
| PAV_r1.0chr3 | 32,315,844 | 18500 | 0.06 |
| PAV_r1.0chr4 | 35,795,787 | 31000 | 0.09 |
| PAV_r1.0chr5 | 29,184,441 | 23500 | 0.08 |
| PAV_r1.0chr6 | 42,573,230 | 26501 | 0.06 |
| PAV_r1.0chr7 | 33,006,389 | 21500 | 0.07 |
| PAV_r1.0chr8 | 32,681,676 | 21000 | 0.06 |
| Total | 305,722,564 | 240501 | 0.07 |
| Anchored ratio (%) | 85.10 | - | - |
| Unanchored | 53,540,899 | - | - |

**Table S7. BUSCO assessment of *de novo* assembly and genome annotation.**

| Type | Assembly | | Annotated genes | |
| --- | --- | --- | --- | --- |
|  | Number | Percentage (%) | Number | Percentage (%) |
| Complete BUSCOs (C) | 1576 | 97.6 | 1519 | 94.1 |
| Complete and single-copy BUSCOs (S) | 1348 | 83.5 | 660 | 40.9 |
| Complete and duplicated BUSCOs (D) | 228 | 14.1 | 859 | 53.2 |
| Fragmented BUSCOs (F) | 16 | 1.0 | 52 | 3.2 |
| Missing BUSCOs (M) | 22 | 1.4 | 43 | 2.7 |
| Total BUSCO groups searched | 1614 | - | 1614 | - |

**Table S8. Statistics of repetitive sequence classification from the PruPse V1.0 genome**

| Type | Length (bp) | Rate (%) |
| --- | --- | --- |
| **Class I: Retrotransposon** | **87,461,604** | **28.61%** |
| **LTR-Retrotransposon** | **81,503,114** | **26.66%** |
| LTR/Copia | 35,013,604 | 11.45% |
| LTR/Gypsy | 44,089,533 | 14.42% |
| LTR-other | 2,399,977 | 0.79% |
| **Non-LTR Retrotransposon** | **5,958,490** | **1.95%** |
| SINE | 29,933 | 0.01% |
| LINE | 5,928,557 | 1.94% |
| **Class II: DNA Transposon** | **46,022,317** | **15.05%** |
| EnSpm/CACTA | 19,647,195 | 6.43% |
| Harbinger | 4,526,287 | 1.48% |
| Helitron | 4,914,023 | 1.61% |
| MuDR | 6,037,067 | 1.97% |
| Tcl/Mariner | 104,399 | 0.03% |
| hAT | 9,343,825 | 3.06% |
| DNA-other | 1,449,521 | 0.47% |
| **Low Complexity** | **18,045** | **0.01%** |
| **Tandem repeat** | **6,162,175** | **2.02%** |
| **Unclassified** | **18,233,304** | **5.96%** |
| **Total content** | **158,306,944** | **51.78%** |

**Table S9. Statistics of gene functional annotation in the PruPse V1.0 genome.**

| Database | Annotated Number | Annotated Percent (%) |
| --- | --- | --- |
| NR | 41,098 | 98.29% |
| Swiss-Prot | 27,838 | 66.58% |
| TAIR | 31,647 | 75.69% |
| MSU | 33151 | 79.29% |
| GO | 12,865 | 30.77% |
| KEGG | 15,949 | 38.15% |
| COG | 35,238 | 84.28% |
| eggNOG | 35,238 | 84.28% |
| Pfam | 27,840 | 66.59% |
| Annotated | 41,105 | 98.31% |
| Total | 41,811 | 100% |

**Table S10. Statistics analysis of HiFi sequencing data for *Prunus pseudocerasus.***

| **Sample** | **Rawdata Base** | **Cleandata Base** | **CCS N50** | **Coverage fold** |
| --- | --- | --- | --- | --- |
| m64154_210507_133818 | 284,120,892,071 | 19,918,328,922 | 18829 | - |
| m64154_210502_095959 | 330,573,887,164 | 22,560,215,345 | 17760 | - |
| Total | 614,694,779,235 | 42,478,544,267 | - | 43 |

**Table S11. Statistical of contig genome assembly based on HiFi data of *Prunus pseudocerasus.***

| Sample ID | Contig | |
| --- | --- | --- |
|  | Length (bp) | Number |
| Total | 993,694,142 | 2,339 |
| Max | 35,130,427 | - |
| N50 | 7,051,323 | 38 |

Note: N50 refers to the size above which 50% of the total length of the sequence assembly can be found.

**Table S12. BUSCO assessment of contig genome assembly based on HiFi data of *Prunus pseudocerasus.***

| Type | Number | Percentage (%) |
| --- | --- | --- |
| Complete BUSCOs (C) | 1591 | 98.60% |
| Complete and single-copy BUSCOs (S) | 56 | 3.50% |
| Complete and duplicated BUSCOs (D) | 1535 | 95.10% |
| Fragmented BUSCOs (F) | 3 | 0.20% |
| Missing BUSCOs (M) | 20 | 1.20% |
| Total BUSCO groups searched | 1375 | - |

**Table S13. Statistics of the contig** **genome assembly of four monoploids.**

|  | **Hap1** | |  | **Hap2** | |  | **Hap3** | |  | **Hap4** | |
| --- | --- | --- | --- | --- | --- | --- | --- | --- | --- | --- | --- |
|  | Length (bp) | Number |  | Length (bp) | Number |  | Length (bp) | Number |  | Length (bp) | Number |
| N10 | 15,118,467 | 2 |  | 18,125,000 | 2 |  | 22,750,411 | 1 |  | 12,967,822 | 2 |
| N20 | 14,776,346 | 3 |  | 13,583,080 | 3 |  | 13,509,545 | 3 |  | 12,484,567 | 3 |
| N30 | 14,439,786 | 5 |  | 10,990,873 | 5 |  | 12,609,809 | 5 |  | 7,606,430 | 5 |
| N40 | 8,716,558 | 7 |  | 9,555,107 | 7 |  | 8,872,718 | 7 |  | 6,245,227 | 8 |
| N50 | 7,840,664 | 10 |  | 6,846,412 | 11 |  | 5,999,568 | 10 |  | 5,432,551 | 12 |
| N60 | 5,865,848 | 14 |  | 6,282,084 | 14 |  | 4,704,141 | 14 |  | 3,530,766 | 16 |
| N70 | 4,344,503 | 19 |  | 4,692,389 | 19 |  | 3,164,709 | 20 |  | 3,035,593 | 22 |
| N80 | 3,624,192 | 25 |  | 3,934,651 | 24 |  | 2,277,947 | 28 |  | 2,477,712 | 29 |
| N90 | 2,153,143 | 33 |  | 2,247,651 | 33 |  | 1,578,156 | 40 |  | 1,451,841 | 39 |
| Total | 246,295,535 | - |  | 237,020,056 | - |  | 225,519,133 | - |  | 192,911,129 | - |
| Contig | - | 65 |  |  | 58 |  |  | 71 |  |  | 69 |

**Table S14. Statistics of haplotype-resolved PruPse assembly**

|  | **Hap1** | |  | **Hap2** | |  | **Hap3** | |  | **Hap4** | |  |
| --- | --- | --- | --- | --- | --- | --- | --- | --- | --- | --- | --- | --- |
|  | Length (bp) | Number |  | Length (bp) | Number |  | Length (bp) | Number |  | Length (bp) | Number |  |
| N10 | 46,520,301 | 1 |  | 44,023,726 | 1 |  | 40,333,243 | 1 |  | 39,864,160 | 1 |  |
| N20 | 34,438,909 | 2 |  | 32,678,172 | 2 |  | 32,145,105 | 2 |  | 39,864,160 | 1 |  |
| N30 | 34,438,909 | 2 |  | 32,678,172 | 2 |  | 32,145,105 | 2 |  | 27,939,879 | 2 |  |
| N40 | 32,548,327 | 3 |  | 32,348,128 | 3 |  | 31,700,875 | 3 |  | 25,622,243 | 3 |  |
| N50 | 28,525,531 | 4 |  | 27,168,469 | 4 |  | 26,332,636 | 4 |  | 24,801,833 | 4 |  |
| N60 | 27,633,680 | 5 |  | 26,814,505 | 5 |  | 26,118,800 | 5 |  | 24,801,833 | 4 |  |
| N70 | 27,259,208 | 6 |  | 26,697,430 | 6 |  | 25,201,163 | 6 |  | 21,017,106 | 5 |  |
| N80 | 26,989,641 | 7 |  | 26,697,430 | 6 |  | 25,201,163 | 6 |  | 19,058,425 | 6 |  |
| N90 | 26,989,641 | 7 |  | 26,602,374 | 7 |  | 24,332,253 | 7 |  | 17,876,795 | 7 |  |
| Total | 246,324,035 | - |  | 237,045,056 | - |  | 225,550,633 | - |  | 192,941,629 | - |  |
| Scaffold | - | 8 |  | - | 8 |  | - | 8 |  | - | 8 |  |

**Table S15**. **Long-terminal-repeat retrotransposon assembly index (LAI) analysis of**

**different genome assemblies in *Prunus* species.**

| Species | LAI | Contig N50 (Mb) |
| --- | --- | --- |
| *P. pseudocerasus*-Hap1 | 20.94 | 7.84 |
| *P. pseudocerasus*-Hap2 | 20.82 | 6.85 |
| *P. pseudocerasus*-Hap3 | 22.07 | 6.00 |
| *P. pseudocerasus*-Hap4 | 21.86 | 5.43 |
| *P. avium*^1^ | 19.68 | 3.25 |
| *P. persica*^2^ | 18.79 | 0.25 |
| *P. armeniaca*^3^ | 16.29 | 1.02 |
| *Prunus pusilliflora*^4^ | 17.35 | 6.00 |
| *P. yedoensis*^5^ | 6.87 | 0.92 |
| *P. domestica*^6^ | 2.27 | 1.74 |

**References:**

1. **Wang, J., Liu, W., Zhu, D., Hong, P., Zhang, S., Xiao, S., Tan, Y., Chen, X., Xu, L., Zong, X.,** **et al.** (2020b). Chromosome-scale genome assembly of sweet cherry (*Prunus avium* L.) cv. Tieton obtained using long-read and Hi-C sequencing. Hortic. Res. **7(1)**:122.
2. **Verde, I., Jenkins, J., Dondini, L., Micali, S., Pagliarani, G., Vendramin, E., Paris, R., Aramini, V., Gazza, L., Rossini, L., et al.（2017）**The Peach v2.0 release: high-resolution linkage mapping and deep resequencing improve chromosome-scale assembly and contiguity**.** BMC Genomics **18(1)**:225.
3. **Jiang, F., Zhang, J., Wang, S., Yang, L., Luo, Y., Gao, S., Zhang, M., Wu, S., Hu, S., Sun, H., et al.** (2019). The apricot (*Prunus armeniaca* L.) genome elucidates Rosaceae evolution and beta-carotenoid synthesis. Hortic. Res. **6**:128.
4. **Jiu, S., Chen, B., Dong, X., Lv, Z., Wang, Y., Yin, C., Xu, Y., Zhang, S., Zhu, J., Wang, J., et al. (2023).** Chromosome-scale genome assembly of *Prunus pusilliflora* provides novel insights into genome evolution, disease resistance, and dormancy release in *Cerasus* L. Hortic. Res. **10(5)**: uhad062.
5. **Baek, S., Choi, K., Kim, G.B., Yu, H.J., Cho, A., Jang, H., Kim, C., Kim, H.J., Chang, K.S., Kim, J.H., et al.** (2018). Draft genome sequence of wild *Prunus yedoensis* reveals massive inter-specific hybridization between sympatric flowering cherries. Genome Biol. **19:**1–17.
6. **Zhebentyayeva, T., Shankar, V., Scorza, R., Callahan, A., Ravelonandro, M., Castro, S., DeJong, T., Saski, C.A., and Dardick, C.** (2019). Genetic characterization of worldwide *Prunus domestica* (plum) germplasm using sequence-based genotyping. Hortic. Res. **6**:12.
